# A Multigene Perspective on the Phylogeny and Evolutionary Relationships of Class Spirotrichea (Ciliophora)

**DOI:** 10.1002/ece3.72668

**Published:** 2025-12-16

**Authors:** Bailin Li, Chunyu Lian, Yongqiang Liu, Zhongming Wang, Xuming Pan, Li Wang

**Affiliations:** ^1^ Key Laboratory of Biodiversity of Aquatic Organisms Harbin Normal University Harbin P.R. China; ^2^ Harbin Mazars Enterprise Management Consulting co. Harbin P.R. China

**Keywords:** Discocephalida, Licnophoria, multigene, phylogeny, Spirotrichea

## Abstract

Despite extensive research, evolutionary relationships within the class Spirotrichea remain contentious due to limitations of single‐gene phylogenies and sparse molecular data. This study provides the first comprehensive phylogenomic framework integrating four molecular markers (SSU‐rRNA, ITS1‐5.8S‐ITS2, LSU‐rRNA, mtSSU‐rRNA), morphological traits, and dual rRNA secondary structures, resolving long‐standing taxonomic conflicts. Key advances include: (1) Critical expansion of underrepresented molecular data: 19 novel mtSSU‐rRNA sequences generated for Spirotrichea, a gene previously scarce in this class, enabling robust phylogenetic reconstruction. (2) Redefinition of major evolutionary lineages: Spirotrichea is restructured into four distinct clades: (i) Subclass Euplotia (excluding Discocephalida); (ii) Subclasses Hypotrichia + Oligotrichia + Order Discocephalida; (iii) Subclasses Protohypotrichia + Phacodinidia; (iv) Subclass Licnophoria as the earliest‐diverging lineage. (3) Resolution of key taxonomic uncertainties: Certesiidae as the sister group of Euplotidae. Discocephalida is reclassified as an evolutionary pivot linking Euplotia and Hypotrichia, evidenced by ITS2 Type 4 architecture and ciliary homologies. *Lynnella semiglobulosa* bridges Tintinnida and Strombidiida, elucidating oligotrich evolution. Licnophoria's basal status is solidified by unique nSSU‐V4 structures homologous to Euplotia, rejecting alternative placements. (4) Ecological drivers of divergence: Niche transitions (e.g., marine‐to‐freshwater shifts in Urostylida) align with phylogenetic topologies, revealing habitat adaptation as a speciation mechanism.

## Graphical abstract

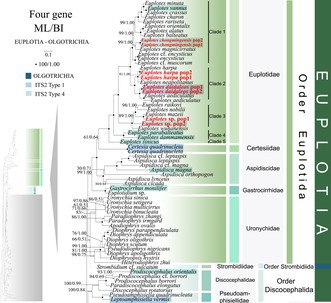

## 1 Introduction

Ciliated protozoa are highly specialized microbial eukaryotes with distinctive structural traits, such as cilia during their life cycle and nuclear dimorphism, which set them apart from other protozoa (Becz and Török 2023; Chi et al. 2024; Kouser et al. 2022; Lyu et al. 2024; Li et al. 2025; Weisse and Montagnes 2022; Wu et al. 2023). According to Lynn (2008), the phylum Ciliophora is divided into 11 classes, although new classes have subsequently been established through the elevation of taxa at subclass or other ranks (e.g., Protocruziea and Mesodiniea), and so on (Gao et al. 2016; Jiang et al. 2024; Rotterová et al. 2020). Among ciliates, the class Spirotrichea is ubiquitous, occurring in nearly every habitat, and is widely regarded as one of the most diverse and taxonomically most challenging assemblages (Liu et al. 2017; Lobban et al. 2002; Sheng et al. 2018). Spirotrichea ciliates are widely distributed across various habitats and often dominate ciliate communities (Abraham et al. 2024; Chu et al. 2025; da Silva and Fernandes 2024; Huber et al. 2025). Their crucial role in carbon cycling within microbial food webs grants them significant ecological importance, as evidenced by the use of *Euplotes* ciliates as indicator species in wastewater treatment (Lawrence and Snyder 1998; Boscaro et al. 2019). Furthermore, the remarkable species diversity within the class Spirotrichea makes it an ideal model for studying ciliate evolution (Hu et al. 2025; Wang et al. 2023; Zhang et al. 2025). Therefore, establishing a robust phylogenetic framework is essential for accurately understanding the evolutionary history of this group. To date, phylogenetic relationships among spirotrich clades and other ciliates have been reconstructed through comparative analysis of ciliary patterns combined with molecular phylogenies based on available DNA sequence data (Dong et al. 2022; Gao et al. 2016; Lian et al. 2023; Lu et al. 2023; Omar et al. 2024; Wang et al. 2025). However, the unresolved phylogenetic relationships within spirotricheans hinder deeper investigations in related biological disciplines. These relationships have undergone multiple revisions, resulting in inconsistent taxonomic schemes (Adl et al. 2012; Corliss 1979; Gao et al. 2016; Lynn 2008; Sheng et al. 2018).

Currently, the class Spirotrichea encompasses six subclasses: Euplotia, Hypotrichia, Oligotrichia, Licnophoria, Protohypotrichia, and Phacodiniidia (Lian et al. 2023; Sheng et al. 2018; Song et al. 2023). The subclass Euplotia represents a morphologically complex and species‐rich ciliate subclass, historically relied on subjective morphological classification (Abraham et al. 2021; Choi et al. 2022; Curds 1975; Lian et al. 2021; Yan et al. 2018). Although molecular markers (e.g., rRNA gene, CO1) now complement traditional approaches, critical uncertainties persist, notably the weak phylogenetic support for discocephalids as sister to euplotids despite their placement in current taxonomic frameworks (Di Giuseppe et al. 2012; Gao et al. 2016; Lian et al. 2023). Subclass‐level sampling is highly uneven, with most species predominantly represented in Hypotrichia (Liao et al. 2025; Liu et al. 2024; Zhao et al. 2025). A plausible explanation is that hypotrichs are widely acknowledged as the most morphologically complex and highly differentiated ciliate assemblage, which appears to justify the concentrated sampling efforts on Hypotrichia. Nevertheless, even at this scale, relationships within Hypotrichia remain poorly resolved, exhibiting pervasive non‐monophyly issues (Foissner and Berger 2021; Gao et al. 2016; Vďačný and Foissner 2021; Yi and Song 2011; Zhang et al. 2022). The taxonomy of Oligotrichia has been systematically revisited in recent research. Through a trinity of evidence integrating multi‐gene sequences, morphological characteristics, and ITS2 structure, Song et al. (2023) established Oligotrichia and Choreotrichia as monophyletic groups, unifying them within the systematic framework of the class Spirotrichea. Phacodiniidia and Protohypotrichia, with shared morphogenetic traits such as poorly differentiated dorsal, ventral ciliature and a relatively large buccal field, are widely regarded as early‐branching groups within Spirotrichea. Limited studies further suggest that Protohypotrichia may encompass independent evolutionary lineages (e.g., *Kiitricha* and *Caryotricha*) (de Calvo Pablo 2010; Lian et al. 2023). Beyond widely studied taxa, there exist ‘forgotten’ taxonomic groups that receive minimal research attention, as exemplified by the subclass Licnophoria.

To achieve more robust phylogenetic reconstructions, studies increasingly leverage multi‐gene sequences or phylogenomic data rather than single gene markers (Leigh et al. 2008; Liu et al. 2024; Rokas et al. 2005; Sun et al. 2021; Yi and Song 2011). Given limited phylogenomic data availability for representative species, we employed a multi‐gene tree approach to reconstruct evolutionary relationships across all six recognized Spirotrichea subclasses. Nuclear rRNA genes, typically referring to three molecular markers (SSU‐rRNA, ITS1‐5.8S‐ITS2, and LSU‐rRNA genes), are among the most widely used genetic markers in phylogenetic studies, providing a rich source of molecular data (Gentekaki et al. 2017). Beyond the SSU‐rRNA gene, the mitochondrial small subunit ribosomal RNA gene (mtSSU‐rRNA) serves as a valuable alternative marker due to its elevated copy number and accelerated evolutionary rate, proving particularly effective for resolving relationships at shallow phylogenetic nodes (Boore and Brown 1998; Dunthorn et al. 2014; Katz et al. 2011; Moore 1995; Rand 2003; Wang et al. 2017).

When morphological and molecular data are in conflict, conserved secondary structures of ribosomal RNA are often employed as supplementary evidence to resolve phylogenetic relationships (Coleman 2005; Strüder‐Kypke et al. 2000; Weimer et al. 2023; Zhang and Vd'ačný 2022). The secondary structures of the nuclear small subunit ribosomal RNA (nSSU‐rRNA) V4 region and internal transcribed spacer 2 (ITS2) each carry key phylogenetic signals through highly conserved helical domains—characterized by structural features such as the E23_8 helix in nuclear SSU‐V4, helices in ITS2 (Wardani et al. 2025; Zhang and Vd'ačný 2022). Although sequence length and composition vary markedly (e.g., mitochondrial V4 region: 64–496 bp, AT‐enriched), the conserved secondary structure landscape, exceeding sequence‐based conservation, directly reflects lineage diversification via signature variations: nSSU‐V4 conformations (single/double helical) and ITS2 terminal loop polymorphisms (Yi and Song 2011; Wardani et al. 2025; Zhang et al. 2019; Zhang and Vd'ačný 2022). These multidimensional molecular‐morphological characteristics provide independent evidence for species identification, cryptic species discovery, and higher‐level phylogenetic reconstruction (Schultz and Wolf 2009; Wang et al. 2017; Zhang et al. 2019).

This study reports 85 novel sequences from 25 populations spanning 18 spirotrichean species. Furthermore, this research reconstructs a comprehensive phylogeny of the class Spirotrichea by integrating three nuclear markers (SSU‐rRNA, ITS1‐5.8S‐ITS2, LSU‐rRNA gene) with mtSSU‐rRNA gene data and morphological characters.

## 2 Material and Methods

### 2.1 Taxon Sampling, Observation, and Terminology

Totally, 25 populations of 18 species assigned to genera *Euplotes*, *Strongylidium*, *Pseudonotohymena*, *Urostyla*, *Pelagohalteria*, *Cyrtohymena*, *Stylonychia*, *Halteria*, *Anteholosticha*, *Oxytricha*, *Urosoma*, and *Paraurostyla* were collected in this study. Specimens of *Oxytricha* and *Urosoma* were isolated from soil samples collected in Zhaodong, China. The remaining species were obtained from several freshwater sites in Heilongjiang, China (Table 1). Soil ciliates were harvested using the non‐flooded Petri dish method under ambient conditions (ca. 25°C). The remaining species were maintained in native habitat water and cultured in Petri dishes at 25°C. Wheat grains provided nutrients in the Petri dishes to stimulate bacterial growth. The protargol staining technique was used to reveal the ciliary pattern (Wilbert 1975). Terminology and classification were mainly according to Gao et al. (2016), Lian et al. (2023) and Lynn (2008).

**TABLE 1:** The sampling sites in this study.

Species	Sampling site	Sampling methods	Species	Sampling site	Sampling methods
*Euplotes* sp. pop1	A freshwater pond, Zhaodong (46°4′38″ N; 125°56′22″ E)	Collected from water using bottle	*Cyrtohymena primicirrata*	Puddle water, Hulan district, Harbin (45°58′3″ N; 126°35′43″ E)	Collected from water using bottle
*Euplotes* sp. pop2	A freshwater pond, Longfeng district, Daqing (46°32′22″ N; 125°10′45″ E)	Collected from water using bottle	*Paraurostyla weissei*	Puddle water, Hulan district, Harbin (45°53′12″ N; 126°35′6″ E)	Collected from water using bottle
*Euplotes daidaleos* pop1	a freshwater pond, Hulan district, Harbin (45°57′5″ N; 126°34′45″ E)	Collected from water using bottle	*Pseudonotohymena antarctica*	Soil by the pond edge, Zhaodong (46°4′52″ N; 125°56′37″ E)	Collected from soil using trowel
*Euplotes daidaleos* pop2	Inland rivers, Xiangfang district, Harbin (45°42′40″ N; 126°38′14″ E)	Collected from water using bottle	*Halteria grandinella*	Inland rivers, Xiangfang district, Harbin (45°42′40″ N; 126°38′14″ E)	Collected from water using bottle
*Euplotes harpa* pop1	A freshwater pond, Longfeng district, Daqing (46°32′22″ N; 125°10′45″ E)	Collected from water using bottle	*Pelagohalteria cirrifera*	Inland rivers, Hulan district, Harbin (45°53′12″ N; 126°35′6″ E)	Collected from water using bottle
*Euplotes harpa* pop2	A freshwater pond, Longfeng district, Daqing (46°32′21″ N; 125°10′45″ E)	Collected from water using bottle	*Urosoma emarginata*	Soil by the pond, Zhaodong (46°4′52″ N; 125°56′37″ E)	Collected from soil using trowel
*Euplotes chongmingensis* pop1	Puddle water, Baoshan district, Shuangyashan (46°33′24″ N; 131°39′10″ E)	Collected from water using bottle	*Oxytricha granulifera* pop1	Puddle water, Datong district, Daqing (45°47′35″ N; 125°32′14″ E)	Collected from water using bottle
*Euplotes chongmingensis* pop2	Puddle water, Baoshan district, Shuangyashan (46°34′30″ N; 131°41′12″ E)	Collected from water using bottle	*Oxytricha granulifera* pop2	a freshwater pond, Zhaodong (46°4′52″ N; 125°56′37″ E)	Collected from water using bottle
*Stylonychia mytilus* pop1	Puddle water, Datong district, Daqing (45°47′35″ N; 125°32′14″ E)	Collected from water using bottle	*Oxytricha granulifera* pop3	Puddle water, Baoshan district, Shuangyashan (46°33′24″ N; 131°39′10″ E)	Collected from water using bottle
*Stylonychia mytilus* pop2	A freshwater pond, Zhaodong (46°4′38″ N; 125°56′22″ E)	Collected from water using bottle	*Oxytricha granulifera* pop4	Puddle water, Hulan district, Harbin (45°53′12″ N; 126°35′6″ E)	Collected from water using bottle
*Stylonychia lemnae*	A freshwater pond, Zhaodong (46°4′38″ N; 125°56′22″ E)	Collected from water using bottle	*Strongylidium mucicola*	Inland rivers, Hulan district, Harbin (45°53′12″ N; 126°35′6″ E)	Collected from water using bottle
*Bakuella subtropica*	Inland rivers, Xiangfang district, Harbin (45°42′40″ N; 126°38′14″ E)	Collected from water using bottle	*Anteholosticha monilata*	Puddle water, Baoshan district, Shuangyashan (42°24′59″ N; 128°6′24″ E)	Collected from water using bottle
*Urostyla grandis*	Puddle water, Baoshan district, Shuangyashan (42°24′59″ N; 128°6′24″ E)	Collected from water using bottle			

### 2.2 DNA Extraction, Amplification, and Sequencing

For each species, 8–10 individual cells were isolated using a stereomicroscope. These cells were rinsed with distilled water to eliminate potential contaminants and subsequently incubated in non‐nutrient distilled water for 6 h to ensure the removal of any residual food particles. The cells were then transferred into an Eppendorf tube, ensuring the liquid volume did not exceed 5 μL. Finally, total genomic DNA was extracted from the cells using the DNeasy Blood & Tissue Kit (QIAGEN, Germany, supplied by Shanghai), strictly following the manufacturer's protocol.

Ten individual primers were utilized for target gene amplification, comprising both forward and reverse oligonucleotides tailored to specific loci (Table 2), with all polymerase chain reaction parameters systematically detailed in Table 3. Following a standardized workflow encompassing purification (TIANGEL Midi Purification Kit, TIANGEN BIOTECH, Beijing), ligation (PMD 18‐T vector cloning system, Takara Biomedicals), and transformation (DH5ɑ chemically competent cells, weidibio, Shanghai) of PCR amplicons, three recombinant colonies per target gene were selected for bidirectional Sanger sequencing by Sangon Biotech (Shanghai). Following stringent screening, a single high‐fidelity sequence per gene locus was selected for downstream phylogenetic analysis. A total of 94 novel molecular markers were characterized from 18 spirotrichean species, comprising 23 SSU‐rRNA, 24 ITS1‐5.8S‐ITS2, 23 LSU‐rRNA, and 19 mtSSU‐rRNA gene sequences (GenBank accession numbers, lengths, and G&C contents are shown in Table 4).

**TABLE 2:** Primers used in the polymerase chain reactions for ciliate SSU‐rRNA, ITS1‐5.8S‐ITS2 rRNA and mtSSUrRNA gene in the present study.

	Primer name	Primer sequence
SSU‐rRNA	Euk‐A	5′‐AAC CTG GTT GAT CCT GCC AGT‐3′ (Medlin et al. 1988)
	Euk‐B	5′‐TGA TCC TTC TGC AGG TTC ACC TAC‐3′ (Medlin et al. 1988)
ITS1‐5.8S‐ITS2 rRNA	5.8S‐F	5′‐GTA GGT GAA CCT GCG GAA GGA TCA TTA‐3′ (Goggin 1994)
	5.8S‐R	5′‐TAC TGA TAT GCT TAA GTT CAG CGG‐3′ (Goggin 1994)
LSU‐rRNA	1F	5′‐ACC CGC TGA ACT TAA GCA T‐3′ (Moreira et al. 2007)
	3R	5′‐AAC CTT GGA GAC CTG AT‐3′ (Moreira et al. 2007)
	FX‐F	5′‐GTA GGT GAA CCT GCA GAA GGA TCA‐3′ (Present study)
	LOR	5′‐GCT ATC CTG AGR GAA ACT TCG‐3′ (Pawlowski 2000)
mtSSU‐rRNA	mtSSU‐NEW‐17‐F1	5′‐GCG GGA RTT TDT DMD AAY GGT GG‐3′ (Zhang et al. 2019)
	mtSSU‐1026‐R1	5′‐GTA CCT TGT GTC AAC TTC ACT C‐3′ (Zhang et al. 2019)

**TABLE 3:** Conditions of PCR reactions used for amplification of five molecular markers analyzed in this study.

Molecular marker	PCR program		
	Initial denaturation	Cycling (denaturation, annealing, extension)	Final extension
SSU‐rRNA gene	94°C/5 min	5 cycles: 94°C/30 s, 56°C/105 s, 72°C/120 s 25 cycles: 94°C/30 s, 60°C/105 s, 72°C/120 s	72°C/10 min
ITS1‐5.8S‐ITS2 rRNA gene	94°C/5 min	35 cycles: 94°C/30 s, 56.1°C/45 s, 72°C/60 s	72°C/10 min
LSU‐rRNA gene	94°C/3 min	35 cycles: 95°C/15 s, 57.2°C/60 s, 72°C/120 s	72°C/10 min
mtSSU‐rRNA gene	94°C/5 min	35 cycles: 95°C/15 s, 52.5°C/60 s, 72°C/120 s	72°C/10 min

**TABLE 4:** New Spirotrichea class genes have been sequenced in this study.

Species	Accession numbers	Lengths (bp)	G&C%	Markers	Species	Accession numbers	Lengths (bp)	G&C%	Markers
*Euplotes* sp. pop1	PX278896	1843	45.31%	SSU rDNA	*Euplotes chongmingensis* pop1	PX248601	1803	44.09%	SSU rDNA
	PX278904	462	41.13%	ITS1‐5.8S‐ITS2		PX278857	498	41.37%	ITS1‐5.8S‐ITS2
	PX278869	1871	46.45%	LSU rDNA		PX278882	1842	48.97%	LSU rDNA
	PX278892	594	22.39%	mtSSU rDNA		PX278831	723	25.17%	mtSSU rDNA
*Euplotes* sp. pop2	PX278906	1928	43.05%	SSU rDNA	*Euplotes chongmingensis* pop2	PX248607	1804	44.18%	SSU rDNA
	PX278905	519	39.31%	ITS1‐5.8S‐ITS2		PX278858	450	41.33%	ITS1‐5.8S‐ITS2
	PX278870	1862	46.29%	LSU rDNA		PX278883	985	51.17%	LSU rDNA
	PX278893	783	24.39%	mtSSU rDNA		PX278832	762	25.98%	mtSSU rDNA
*Euplotes daidaleos* pop1	PX248602	1822	43.19%	SSU rDNA	*Stylonychia mytilus* pop1	PX248612	1694	45.34%	SSU rDNA
	PX278851	462	41.13%	ITS1‐5.8S‐ITS2		PX278863	474	45.57%	ITS1‐5.8S‐ITS2
	PX278877	1807	47.04%	LSU rDNA		PX278888	958	47.29%	LSU rDNA
	PX278826	685	21.17%	mtSSU rDNA		PX278836	844	29.98%	mtSSU rDNA
*Euplotes daidaleos* pop2	PX248603	1827	43.35%	SSU rDNA	*Stylonychia mytilus* pop2	PX248604	1700	45.24%	SSU rDNA
	PX278852	467	40.90%	ITS1‐5.8S‐ITS2		PX278853	469	45.42%	ITS1‐5.8S‐ITS2
	PX278878	1802	47.17%	LSU rDNA		PX278879	964	47.82%	LSU rDNA
	PX278827	682	19.79%	mtSSU rDNA		PX278828	841	29.85%	mtSSU rDNA
*Euplotes harpa* pop1	PX278897	1825	42.58%	SSU rDNA	*Urosoma emarginata*	PX248611	1708	45.43%	SSU rDNA
	PX278848	451	45.68%	ITS1‐5.8S‐ITS2		PX278862	474	47.68%	ITS1‐5.8S‐ITS2
	PX278874	1777	47.05%	LSU rDNA		PX278887	956	48.95%	LSU rDNA
	PX278824	656	25.76%	mtSSU rDNA		PX278891	838	33.53%	mtSSU rDNA
*Halteria grandinella*	PX248605	1692	45.98%	SSU rDNA	*Stylonychia lemnae*	PX278854	519	45.86%	ITS1‐5.8S‐ITS2
	PX278829	667	35.38%	mtSSU rDNA		PX278823	842	29.93%	mtSSU rDNA
*Euplotes harpa* pop2	PX278899	1859	42.76%	SSU rDNA	*Paraurostyla weissei*	PX248613	1711	45.65%	SSU rDNA
	PX278850	452	45.13%	ITS1‐5.8S‐ITS2		PX278864	488	46.52%	ITS1‐5.8S‐ITS2
	PX278876	1792	47.21%	LSU rDNA		PX278889	962	49.17%	LSU rDNA
	PX278825	664	25.30%	mtSSU rDNA		PX278837	954	26.31%	mtSSU rDNA
*Anteholosticha monilata*	PX248614	1703	45.04%	SSU rDNA	*Pseudonotohymena antarctica*	PX278901	1721	45.32%	SSU rDNA
	PX278865	476	46.22%	ITS1‐5.8S‐ITS2		PX278846	488	43.85%	ITS1‐5.8S‐ITS2
	PX278890	957	46.92%	LSU rDNA		PX278872	956	48.85%	LSU rDNA
	PX278838	923	23.29%	mtSSU rDNA	*Oxytricha granulifera* pop2	PX248608	1709	44.82%	SSU rDNA
*Oxytricha granulifera* pop1	PX248606	1687	44.87%	SSU rDNA		PX278859	386	46.89%	ITS1‐5.8S‐ITS2
	PX278856	463	46.44%	ITS1‐5.8S‐ITS2		PX278884	1052	48.47%	LSU rDNA
	PX278881	962	49.06	LSU rDNA		PX278833	890	30.22%	mtSSU rDNA
	PX278830	889	30.48%	mtSSU rDNA	*Oxytricha granulifera* pop4	PX248610	1703	44.98%	SSU rDNA
*Oxytricha granulifera* pop3	PX248609	1718	44.94%	SSU rDNA		PX278861	475	46.53%	ITS1‐5.8S‐ITS2
	PX278860	475	46.53%	ITS1‐5.8S‐ITS2		PX278886	962	48.65%	LSU rDNA
	PX278885	963	48.81%	LSU rDNA		PX278835	889	30.26%	mtSSU rDNA
	PX278834	925	30.81%	mtSSU rDNA	*Pelagohalteria cirrifera*	PX278895	1705	46.39%	SSU rDNA
*Cyrtohymena primicirrata*	PX278898	1701	45.56%	SSU rDNA		PX278902	469	50.32%	ITS1‐5.8S‐ITS2
	PX278849	479	45.72%	ITS1‐5.8S‐ITS2		PX278871	970	51.44%	LSU rDNA
	PX278875	977	49.64%	LSU rDNA	*Urostyla grandis*	PX278894	1689	43.81%	SSU rDNA
*Strongylidium mucicola*	PX278900	1710	45.43%	SSU rDNA		PX278847	473	41.01%	ITS1‐5.8S‐ITS2
	PX278903	465	46.88%	ITS1‐5.8S‐ITS2		PX278873	966	46.27%	LSU rDNA
	PX278868	965	49.02%	LSU rDNA					
*Bakuella subtropica*	PX278855	471	42.25%	ITS1‐5.8S‐ITS2					
	PX278880	959	45.26%	LSU rDNA					

### 2.3 Datasets and Alignments

The phylogenetically structured dataset encompasses representative taxa spanning all subclasses of spirotrichean. This consolidated sequence collection integrates novel molecular profiles generated in this study with publicly archived references sourced from GenBank, all associated accession numbers being systematically cross‐referenced in Figures 3, 4, 5, 6, 7, 8, 9, 10. Sequences exhibiting ambiguous morphological attribution, unverifiable origins, or inadequate length were rigorously excluded to ensure phylogenetic reliability. Five datasets were engineered: (i) SSU‐rRNA gene: SSU‐rRNA gene sequences included representatives of 167 taxa (1779 positions); (ii) ITS1‐5.8 s‐ITS2: ITS1‐5.8S‐ITS2 rRNA sequences of 142 taxa (876 positions); (iii) LSU‐rRNA gene sequences of 133 taxa (1864 positions); (iv) mtSSU‐rRNA gene sequences of 32 taxa (771 positions); (v) Four‐genes: concatenation of the aligned SSU‐rRNA, ITS1‐5.8S‐ITS2 rRNA, LSU‐rRNA, and mtSSU‐rRNA from datasets i–v (171 taxa, 4670 positions). For species with only the complete mitochondrial genome available in public databases, the target gene's coordinates were identified using the “small subunit ribosomal RNA” annotation in the GenBank record. Following this, all extracted sequences were verified for accuracy using a BLASTN search. Sequence alignments were performed using MUSCLE v3.8, followed by manual curation of ambiguous regions (e.g., terminal gaps, hypervariable loops) via BioEdit 7.0.1 (Hall 1999). Two colpodean taxa, *Colpoda inflata* and *Paracolpoda steini*, were designated as outgroups across all datasets based on their established phylogenetic position basal to Spirotrichea.

### 2.4 Phylogenetic Analyses

Maximum likelihood (ML) analyses were performed on the aforementioned five sequence alignments using RAxML‐HPC2 v8.2.12 (Stamatakis 2014) via the CIPRES Science Gateway platform under the GTRGAMMA model (Miller et al. 2010), with 1000 bootstrap replicates. Bayesian inference (BI) analyses employed MrBayes v3.2.7 (Ronquist et al. 2012) through the XSEDE environment on the same CIPRES platform using the GTR + I + G model selected by the Akaike Information Criterion (AIC) in MrModeltest v2.2 (Nylander 2004). Maximum likelihood analyses implemented a rapid non‐parametric bootstrap procedure with 1000 replicates. Bayesian inference employed four Markov chains running for 10,000,000 generations, sampling trees at 100‐generation intervals. Burn‐in was set at 25% of sampled trees prior to consensus tree construction. Resultant phylogenies were visualized using MEGA 7 (Kumar et al. 2016).

### 2.5 Sequence Analyses and Putative Secondary Structure Modeling

Secondary structures of ITS2 and nSSUr‐V4 were predicted using default settings on the Mfold web server (http://www.unafold.org/mfold/applications/rna‐folding‐form.php) (Weimer et al. 2023; Zuker 2003), employing the respective reference sequences of *Pseudoamphisiella quadrinucleata* (GQ246484) and *Euplotes sinicus* (FJ423448). RNA secondary structures were visualized using RnaViz 2.0 to optimize clarity and schematic representation (De Rijk and De Wachter 1997). The phylogenetic subtree of the Four gene clade, comprising taxa with predicted ITS2 secondary structures, was extracted, visualized, and customized using the Interactive Tree of Life (iTOL) web platform (https://itol.embl.de/).

## 3 Results

### 3.1 Phylogenies Reconstructed From Concatenated Sequence Data

**FIGURE 1:**
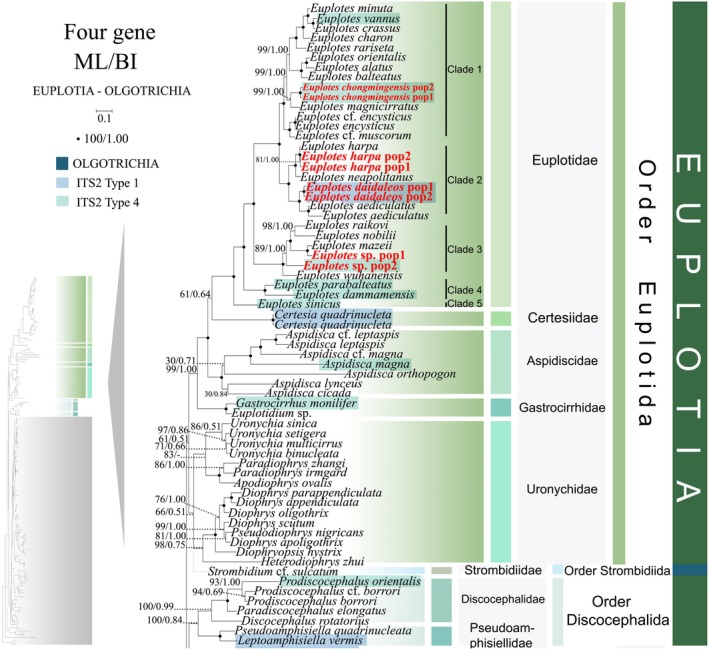
The maximum‐likelihood (ML) tree based on the concatenated genes (SSU‐rRNA, ITS1‐5.8S‐ITS2 rRNA, LSU‐rRNA, and mtSSU‐rRNA genes) of six subclasses of the class Spirotrichea. The lower left part of the image shows a full view of the tree, and the colored part stands for a section of the original image. Newly sequenced species are highlighted in red. The supports for nodes are indicated as follows: ML bootstraps/BI posterior probability. The symbol “‐” denotes inconsistency in topology between the Bayesian and ML trees. Fully supported (100%/1.00) clades are marked with solid circles. “*” at nodes indicates the support values < 30%/0.5 (ML/BI). The scale bar corresponds to 0.1 expected substitutions per site. The branch leading to *Strombidium* cf. *sulcatum* is shown as a gray dashed line due to its unstable position, likely an artifact of Long‐Branch Attraction (LBA) caused by incomplete sequence data. See the main text for details.

**FIGURE 2:**
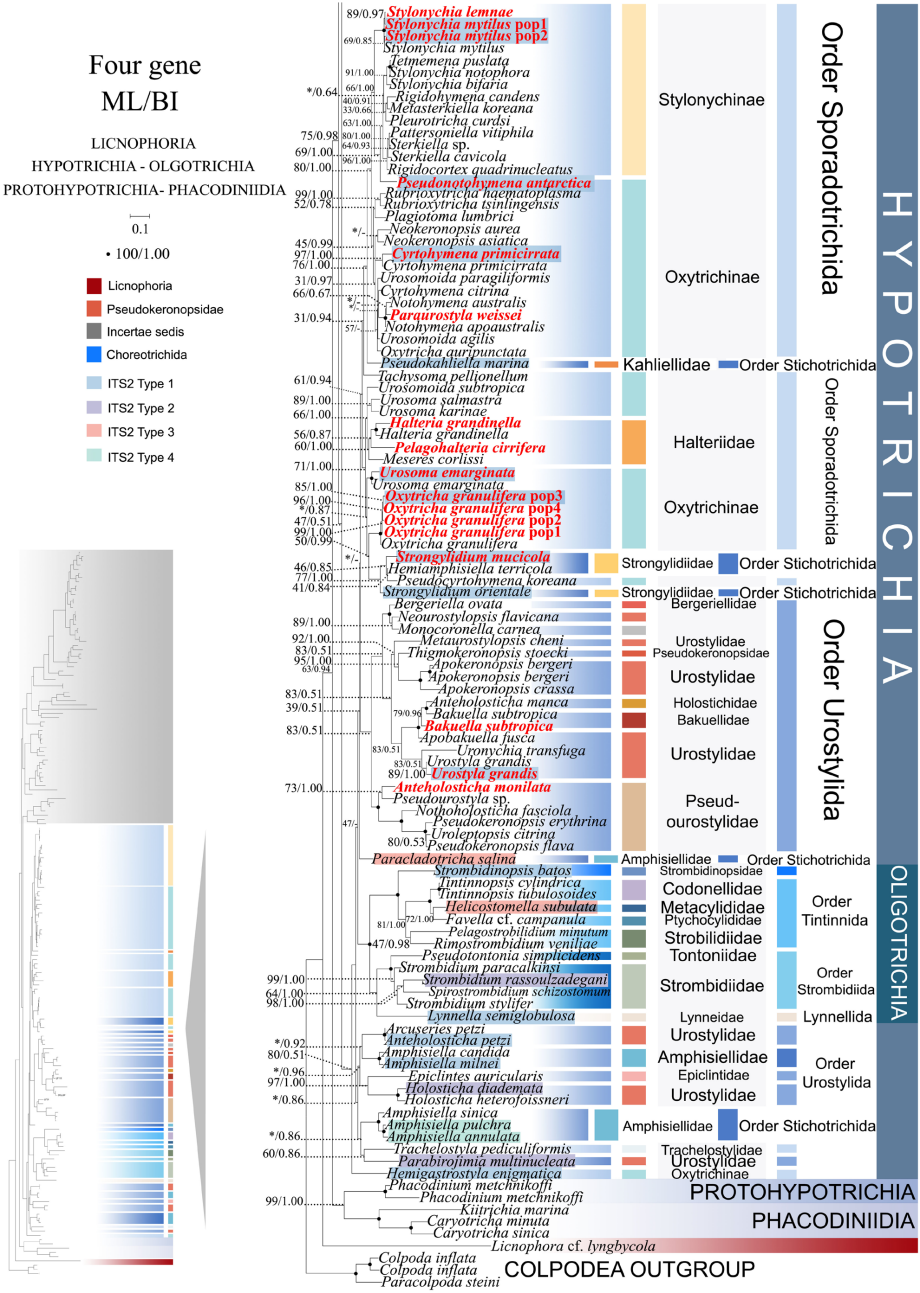
The maximum‐likelihood (ML) tree based on the concatenated genes (SSU‐rRNA, ITS1‐5.8S‐ITS2 rRNA, LSU‐rRNA, and mtSSU‐rRNA genes) of six subclasses of the class Spirotrichea. The lower left part of the image shows a full view of the tree, and the colored part stands for a section of the original image. Newly sequenced species are highlighted in red. The supports for nodes are indicated as follows: ML bootstraps/BI posterior probability. The symbol “‐” denotes inconsistency in topology between the Bayesian and ML trees. Fully supported (100%/1.00) clades are marked with solid circles. “*” at nodes indicates the support values < 30%/0.5 (ML/BI). The scale bar corresponds to 0.1 expected substitutions per site.

Given topological congruence between maximum likelihood (ML) and Bayesian inference (BI) analyses, Figures 1 and 2 present ML phylogenies annotated with nodal support metrics from both methods (ML‐bootstrap values/BI posterior probabilities). To clarify the overall phylogenetic structure, schematic trees summarizing relationships at the order level and family level are provided (Figure 13). Within the class Spirotrichea, phylogenies reconstructed from multi‐gene datasets delineate four primary lineages: (I) Subclass Euplotia and *Strombidium* cf. *sulcatum* (excluding order Discocephalida); (II) Subclasses Hypotrichia and Oligotrichia (encompassing Discocephalida); (III) Subclasses Protohypotrichia and Phacodiniidia; (IV) Subclass Licnophoria (Figures 1 and 2). The subclass Euplotia contains two orders with seven families: Euplotida (families Euplotidae, Certesiidae, Aspidiscidae, Gastrocirrhidae, and Uronychidae) and Discocephalida (Discocephalidae, Pseudoamphisiellidae) (Figure 1). *Euplotes sinicus* occupies the basal position within a monophyletic clade comprising all analyzed *Euplotes* species (supported by 100% ML, 1.00 BI). The family Certesiidae forms a sister clade to Euplotidae (61% ML, 0.64 BI), with this combined lineage successively sister to: (1) Aspidiscidae (100% ML, 1.00 BI); (2) Gastrocirrhidae (99% ML, 1.00 BI); and ultimately (3) Uronychidae + *Strombidium* cf. *sulcatum* (66% ML, 0.51 BI; Figure 1). Notably, Discocephalidae and Pseudoamphisiellidae form a reciprocally monophyletic sister clade (100% ML/0.84 BI), which comprises the order Discocephalida (Figure 1). Contrary to expectations, this order does not cluster with Euplotida but occupies a basal position relative to subclasses Hypotrichia and Oligotrichia (47% ML, 0.51 BI) (Figures 1 and 2).

Within the subclass Hypotrichia, phylogenetic analyses rejected the monophyly of three orders: Sporadotrichida, Stichotrichida, and Urostylida. *Trachelostyla pediculiformis* (Trachelostylidae), *Pseudocyrtohymena koreana* (Oxytrichinae), and *Hemigastrostyla enigmatica* (Oxytrichinae) occupy phylogenetically distant positions from the majority of Sporadotrichida species, scattered across the subclass Hypotrichia. *Trachelostyla pediculiformis* formed a sister clade with *Parabirojimia multinucleata* (Urostylida, Urostylidae) with moderate support (60% ML, 0.86 BI). *Pseudocyrtohymena koreana* clustered as sister to *Hemiamphisiella terricola* (Stichotrichida, Strongylidiidae) under weaker nodal support (41% ML, 0.84 BI). *Hemigastrostyla enigmatica* forms a sister clade to the entire evolutionary assemblage comprising subclasses Hypotrichia and Oligotrichia (80% ML, 0.51 BI). The order Stichotrichida exhibits a polyphyletic distribution across the subclass Hypotrichia, comprising five primary evolutionary lineages:(1) *Pseudokahliella marina*, (2) *Strongylidium mucicola* + *Hemiamphisiella terricola* + *Strongylidium orientale* (77% ML, 1.00 BI), (3) *Paracladotricha salina*, (4) *Amphisiella candida* + *A. milnei* (ML 100%, BI 1.00), and (5) *Amphisiella sinica* + *A. pulchra* + *A. annulata* (100% ML, 1.00 BI). Phylogenetic analyses delineated four Urostylida lineages: (1) a 21‐species core clade (83% ML, 0.51 BI), (2) a clade of two *Amphisiella* spp. (100% ML, 1.00 BI), (3) *Epiclintes auricularis +* two *Holosticha* spp. (97% ML, 1.00 BI), and (4) *Parabirojimia multinucleata* as a singleton lineage. The subclass Oligotrichia is nested within Hypotrichia and comprises four orders: the clade Choreotrichida + Tintinnida is sister to Strombidiida + Lynnellida (100% ML, 1.00 BI), with both lineages forming reciprocally monophyletic sister groups (100% ML, 1.00 BI; 64% ML, 1.00 BI). The subclasses Protohypotrichia and Phacodiniidia form reciprocally monophyletic sister clades (99% ML, 1.00 BI), occupying a basal position relative to the subclasses Euplotia, Hypotrichia, and Oligotrichia (63% ML, 0.94 BI). The subclass Licnophoria, represented by *Licnophora* cf. *lyngbycola*, diverged basally as sister to all other subclasses. Newly sequenced two *Euplotes chongmingensis* populations cluster together with full support. Two Daqing populations of *E. harpa* cluster with previously sequenced ones with full support. *Euplotes* sp. pop1 clusters with *E*. *mazeii* with full support. *Euplotes* sp. pop2 forms a sister clade to four *Euplotes* species with moderate support (89% ML, 1.00 BI). Newly sequenced four Harbin populations of *Oxtricha granulifera* (99% ML, 1.00 BI) cluster with *O. granulifera* with full support. One Harbin population of *Bakuella subtropica* groups with *B. subtropica* *+ Anteholosticha manca* with 79% ML, 0.96 BI.

### 3.2 Phylogenetic Reconstructed From SSU‐rRNA Gene, ITS1‐5.8S‐ITS2 Region and LSU‐rRNA Gene Sequences

**FIGURE 3:**
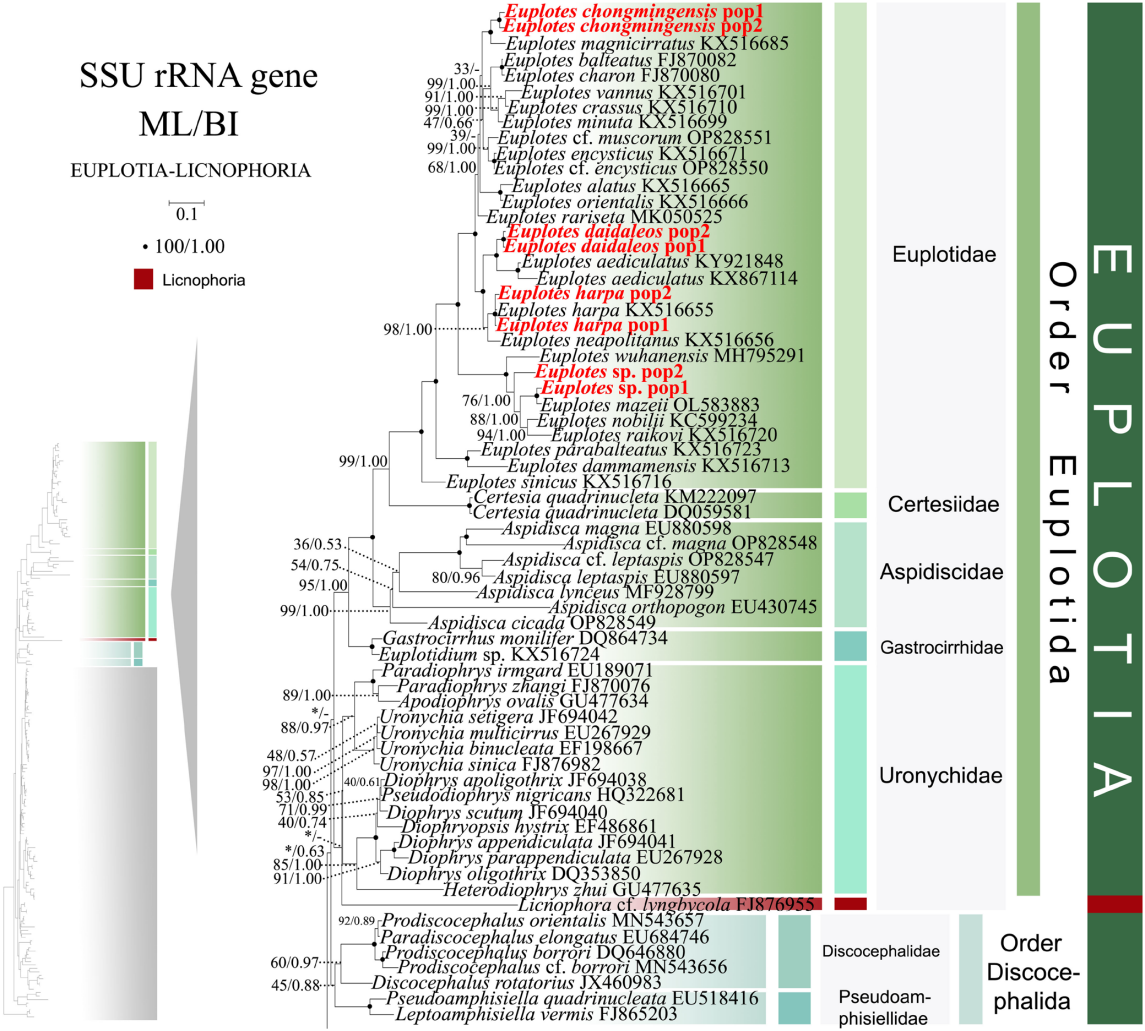
The maximum‐likelihood (ML) tree based on the SSU‐rRNA gene for the class Spirotrichea. The lower left part of the image shows a full view of the tree, and the colored part stands for a section of the original image. Newly sequenced species are highlighted in red. The support for nodes are indicated as follows: ML bootstraps/BI posterior probability. “‐” indicate mismatch in topology between Bayesian and ML trees. Fully supported (100%/1.00) clades are marked with solid circles. “*” at nodes indicates the support values < 30%/0.5 (ML/BI). The long clades has been shortened, as shown by “//”, and the other clades are drawn to scale. The scale bar corresponds to 0.1 expected substitutions per site.

**FIGURE 4:**
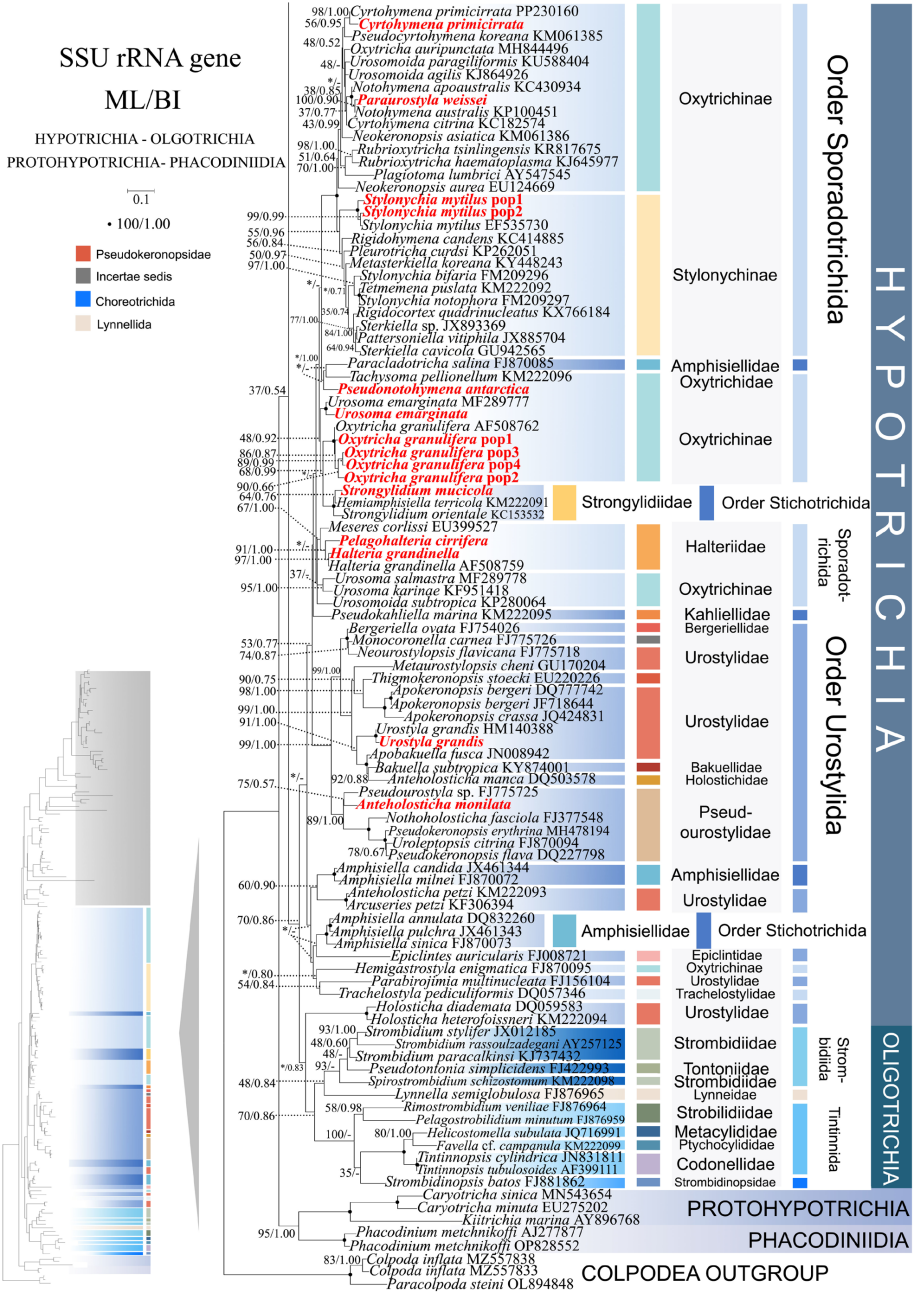
The maximum‐likelihood (ML) tree based on the SSU‐rRNA gene for the class Spirotrichea. The lower left part of the image shows a full view of the tree, and the colored part stands for a section of the original image. Newly sequenced species are highlighted in red. The supports for nodes are indicated as follows: ML bootstraps/BI posterior probability. “‐” indicate mismatch in topology between Bayesian and ML trees. Fully supported (100%/1.00) clades are marked with solid circles. “*” at nodes indicates the support values < 30%/0.5 (ML/BI). The long clades has been shortened, as shown by “//”, and the other clades are drawn to scale. The scale bar corresponds to 0.1 expected substitutions per site.

Phylogenetic reconstruction using SSU‐rRNA sequences revealed topological similarity to concatenated trees (Figures 1 and 2), whereas notable discrepancies emerged in the phylogenetic assignments of several taxa. In the SSU‐rRNA gene phylogeny (Figure 3), the order Discocephalida occupies a phylogenetically distant position from the subclasses Hypotrichia and Oligotrichia, instead forming a sister clade to Euplotida. Conversely, concatenated analyses (Figures 1 and 2) resolve Discocephalida as closely affiliated with Hypotrichia and Oligotrichia. *Licnophora* cf. *lyngbycola* (subclass Licnophoria) and Uronychidae species (order Euplotida) form a sister clade in the SSU‐rRNA phylogeny (Figure 3), whereas in concatenated analyses (Figures 1 and 2) they resolve as sister to the subclasses Euplotia, Hypotrichia, and Oligotrichia. Within subclass Hypotrichia, *Trachelostyla pediculiformis* forms a sister clade with *Parabirojimia multinucleata* (Urostylida, Urostylidae) and then clusters with *Hemiamphisiella terricola* (Figure 4). The subclass Oligotrichia no longer nests within Hypotrichia but instead forms a weakly supported sister clade with two *Holosticha* species, subsequently forming a sister group to Hypotrichia with moderate statistical support (70% ML, 0.86 BI) (Figure 4). The phylogenetic positions of all newly sequenced taxa in the SSU‐rRNA gene tree exhibit robust topological congruence with their placements in the concatenated phylogeny.

**FIGURE 5:**
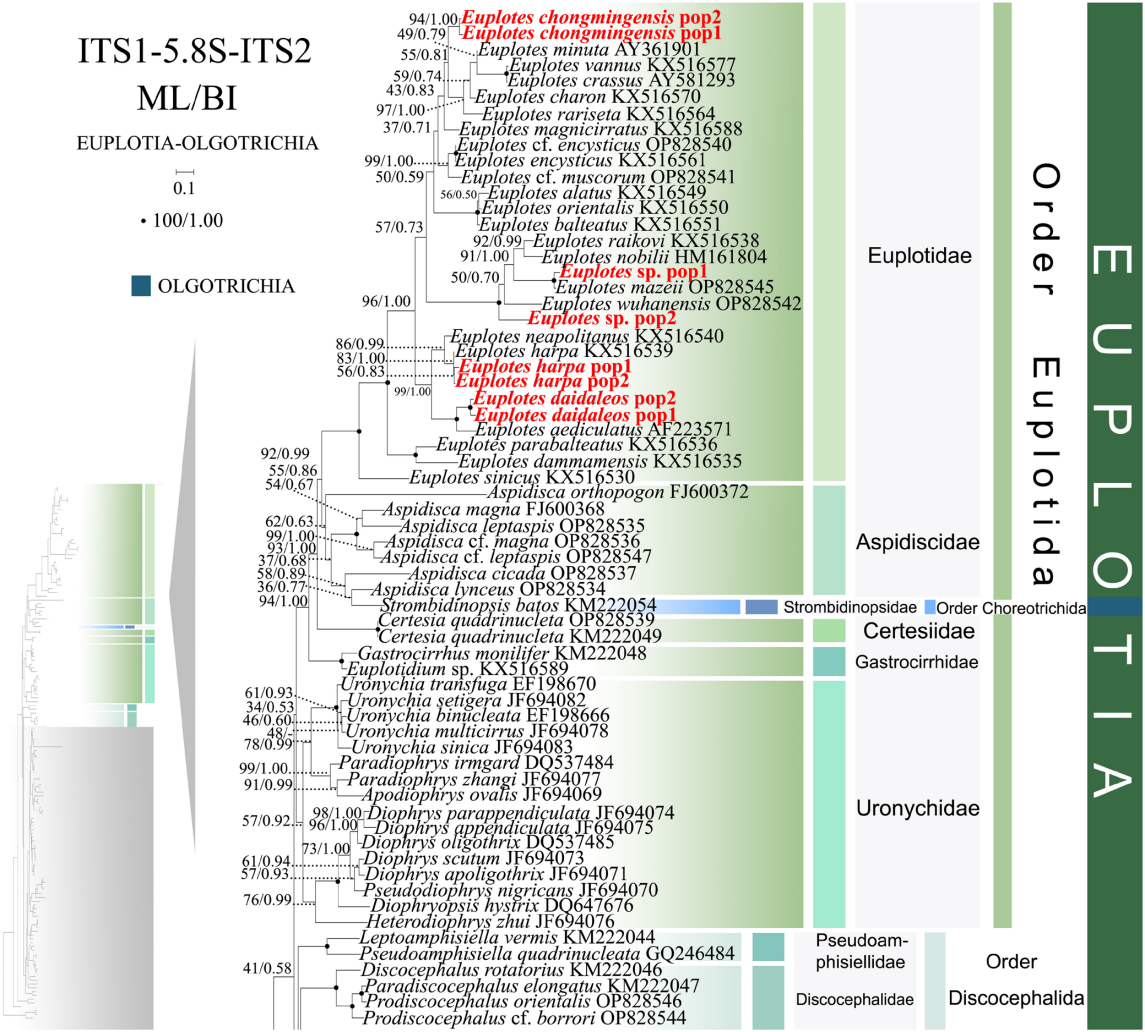
The maximum‐likelihood (ML) tree based on the ITS1‐5.8S‐ITS2 rRNA gene for the class Spirotrichea. The lower left part of the image shows a full view of the tree, and the colored part stands for a section of the original image. Newly sequenced species are highlighted in red. The supports for nodes are indicated as follows: ML bootstraps/BI posterior probability. “‐” indicate mismatch in topology between Bayesian and ML trees. Fully supported (100%/1.00) clades are marked with solid circles. “*” at nodes indicates the support values < 30%/0.5 (ML/BI). The scale bar corresponds to 0.1 expected substitutions per site.

**FIGURE 6:**
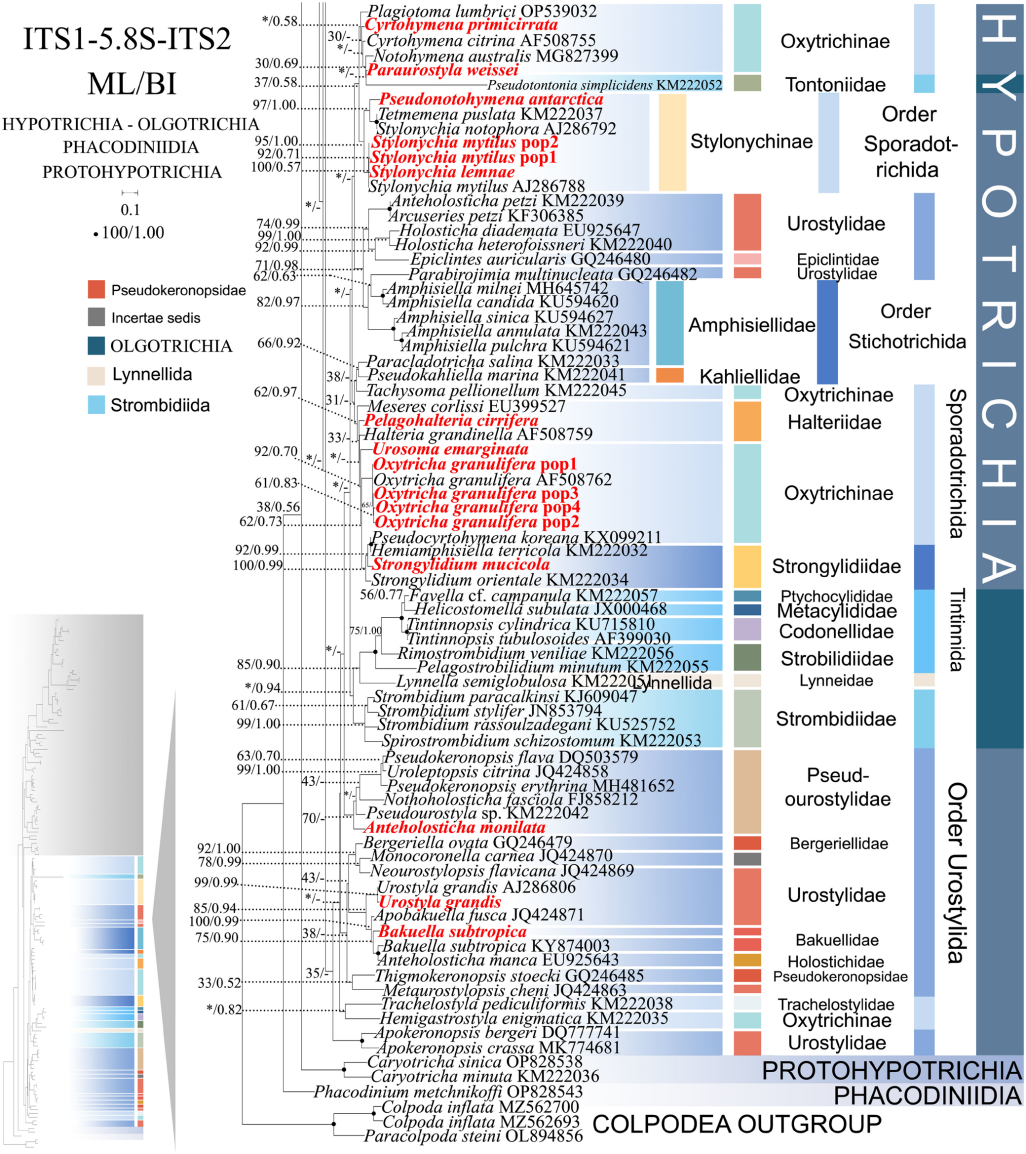
The maximum‐likelihood (ML) tree based on the ITS1‐5.8S‐ITS2 rRNA gene for the class Spirotrichea. The lower left part of the image shows a full view of the tree, and the colored part stands for a section of the original image. Newly sequenced species are highlighted in red. The supports for nodes are indicated as follows: ML bootstraps/BI posterior probability. “‐” indicate mismatch in topology between Bayesian and ML trees. Fully supported (100%/1.00) clades are marked with solid circles. “*” at nodes indicates the support values < 30%/0.5 (ML/BI). The scale bar corresponds to 0.1 expected substitutions per site.

In comparison to the concatenated tree (Figures 1 and 2), the ITS1‐5.8S‐ITS2 rRNA tree (Figures 5 and 6) shows minor differences in its topologies. Primary topological variations were documented within the subclass Oligotrichia. The order Choreotrichida, represented by *Strombidinopsis batos*, diverges from the subclass Oligotrichia and forms a weakly supported sister clade with *Aspidisca lynceus* (family Aspidiscidae, subclass Euplotia) (36% ML, 0.77 BI). However, in the concatenated tree (Figure 2), the order Choreotrichida forms a sister clade with the order Tintinnida with full support. *Pseudotontonia simplicidens* diverges from the subclass Oligotrichia and forms a weakly supported sister clade with *Paraurostyla weissei*. In contrast, concatenated analyses resolve *P*. *simplicidens* at the basal node of the order Strombidiida (100% ML, 1.00 BI). The order Lynnellida forms a sister clade with Tintinnida (85% ML, 0.90 BI) (Figure 6), contrasting with its phylogenetic displacement in concatenated analyses where it clusters as sister to Strombidiida (64% ML, 0.90 BI) (Figure 2).

**FIGURE 7:**
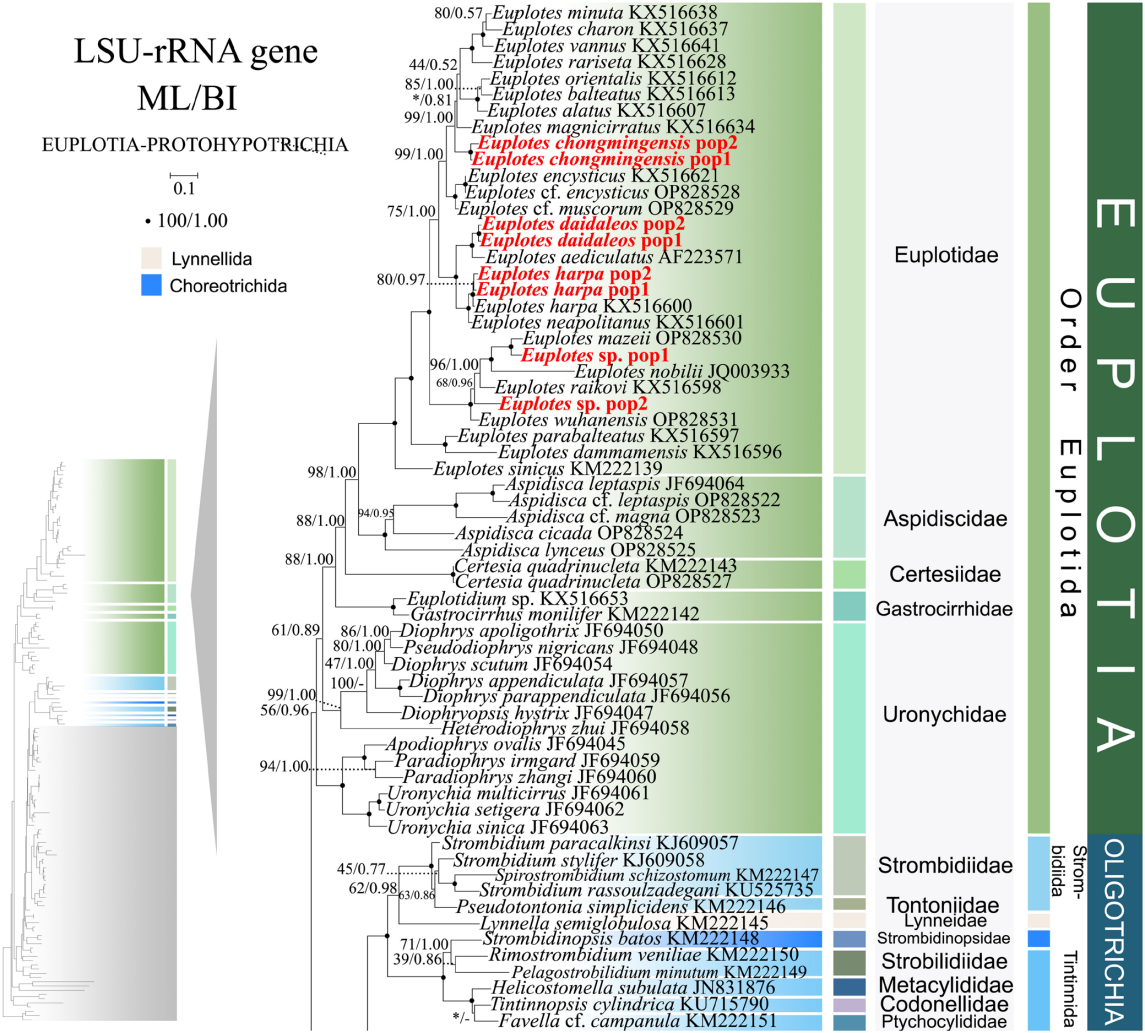
The maximum‐likelihood (ML) tree, based on the LSU‐rRNA gene. The lower left part of the image shows a full view of the tree, and the colored part stands for a section of the original image. Newly sequenced species are highlighted in red. The supports for nodes are indicated as follows: ML bootstraps/BI posterior probability. “‐” indicate mismatch in topology between Bayesian and ML trees. Fully supported (100%/1.00) clades are marked with solid circles. “*” at nodes indicates the support values < 50%/0.5 (ML/BI). The scale bar corresponds to 0.1 expected substitutions per site.

**FIGURE 8:**
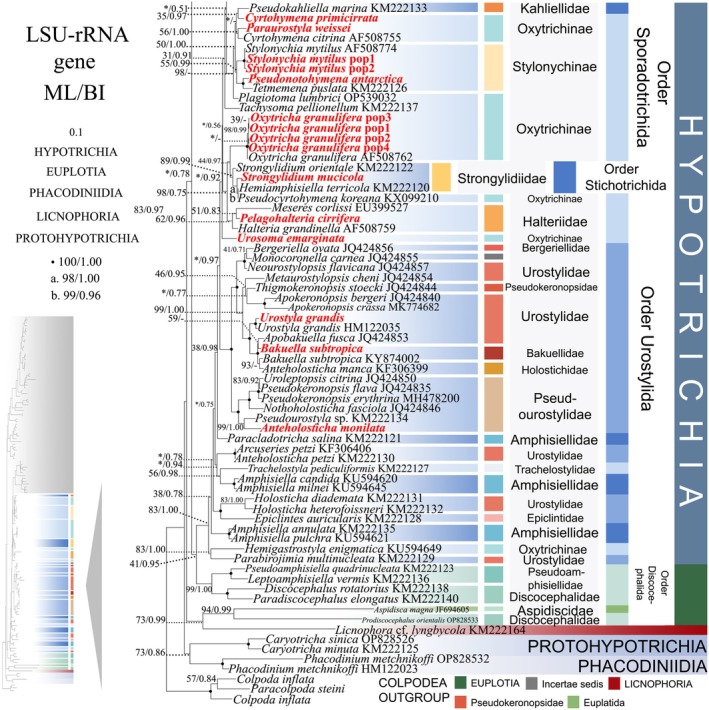
The maximum‐likelihood (ML) tree, based on the LSU‐rRNA gene. The lower left part of the image shows a full view of the tree, and the colored part stands for a section of the original image. Newly sequenced species are highlighted in red. The supports for nodes are indicated as follows: ML bootstraps/BI posterior probability. “‐” indicate mismatch in topology between Bayesian and ML trees. Fully supported (100%/1.00) clades are marked with solid circles. “*” at nodes indicates the support values < 50%/0.5 (ML/BI). The scale bar corresponds to 0.1 expected substitutions per site.

Constrained by limited sequence availability, the LSU‐rRNA gene phylogeny contains fewer operational taxonomic units (OTUs) compared to ITS1‐5.8S‐ITS2 and SSU‐rRNA reconstructions. Nevertheless, it exhibits minor topological deviations (see Figures 7 and 8). In the LSU‐rRNA gene phylogeny, the two lineages of Uronychidae no longer form sister clades. Instead, a novel composite lineage comprising three *Uronychia* species, two *Paradiophrys* species, and one *Apodiophrys* species occupies a basal position within the subclass Euplotia (56% ML, 0.96 BI) (Figure 7). The order Lynnellida forms a sister clade with Tintinnida (62% ML, 0.98 BI), while the order Choreotrichida clusters as sister to two Strombidiida species (*Rimstrombidium veniliae* and *Pelagostrobilidium minutum*) (71% ML, 1.00 BI) (Figure 7). The order Discocephalida occupies a basal position to the subclasses Hypotrichia and Oligotrichia (41% ML, 0.95 BI). Notably, *Aspidisca magna* (Aspidiscidae, Euplatida) and *Prodiscocephalus orientalis* (Discocephalidae, Euplatida) diverged from their ancestral clades, forming a sister lineage (94% ML, 0.99 BI) that subsequently clustered with *Licnophora* cf. *lyngbycola* (73% ML, 0.99 BI) (Figure 8). This novel assemblage is positioned peripherally to the three subclasses.

### 3.3 Phylogenetic Reconstruction From mtSSU‐rRNA Sequences

**FIGURE 9:**
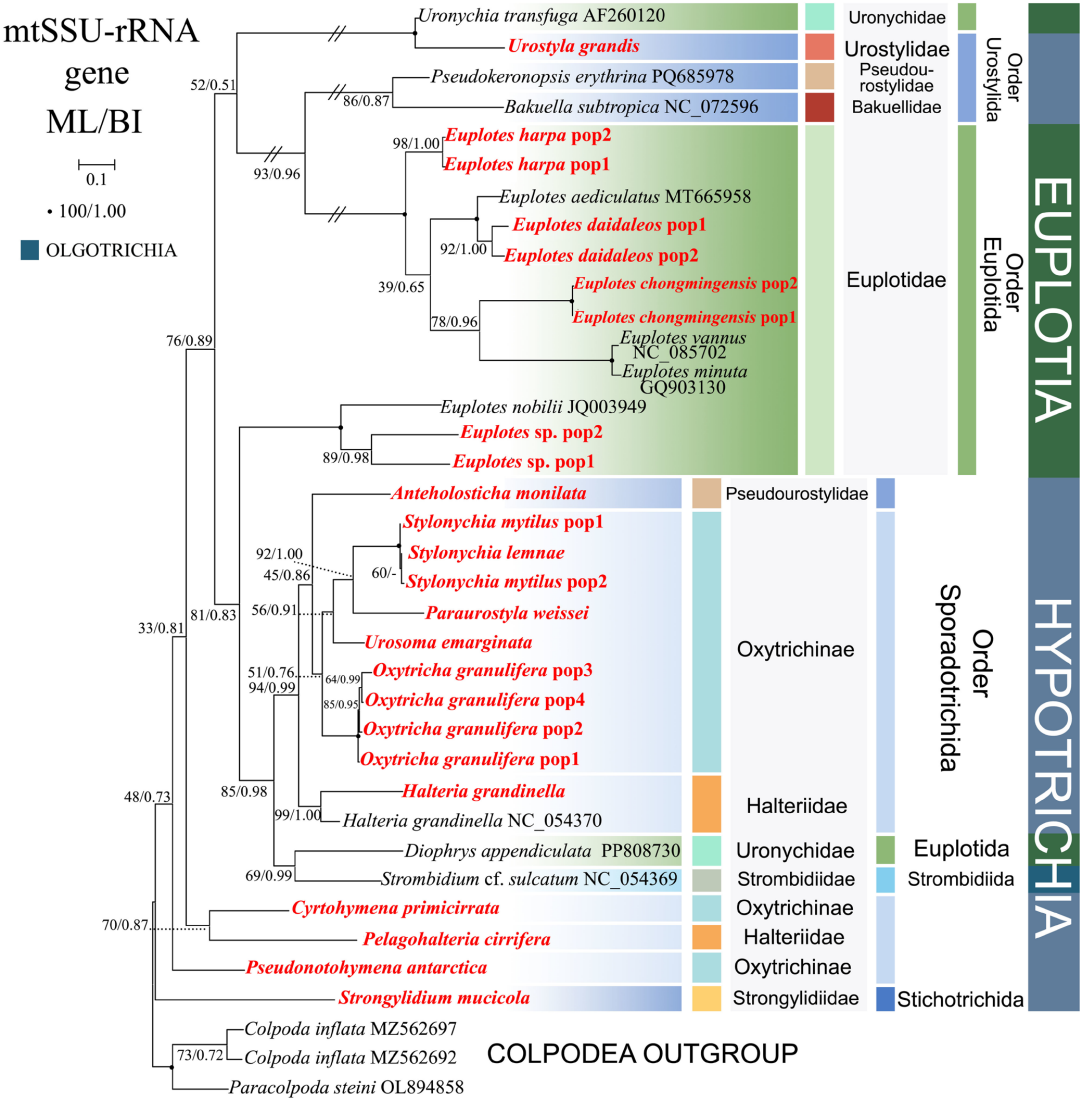
The maximum‐likelihood (ML) tree, based on the mtSSU‐rRNA gene. Newly sequenced species are highlighted in red. The supports for nodes are indicated as follows: ML bootstraps/BI posterior probability. “‐” indicate mismatch in topology between Bayesian and ML trees. Fully supported (100%/1.00) clades are marked with solid circles. “*” at nodes indicates the support values < 50%/0.5 (ML/BI). The long clades has been shortened, as shown by “//”, and the other clades are drawn to scale. The scale bar corresponds to 0.1 expected substitutions per site.

The mtSSU‐rRNA gene tree comprises only sequences of subclasses Euplotia, Hypotrichia, and Oligotrichia. The three subclasses collectively exhibit non‐monophyletic evolutionary patterns. The subclass Euplotia resolves into three phylogenetically distinct lineages: (1) *Uronychia transfuga* and *Diophrys appendiculata*; (2) a clade comprising nine *Euplotes* species; (3) a clade including *Euplotes nobilii*, *Euplotes* sp. pop1, and *Euplotes* sp. pop2. The subclass Oligotrichia is represented by *Strombidium* cf. *sulcatum*, which forms a sister clade with *Uronychia transfuga* and *Diophrys appendiculata*.

The subclass Hypotrichia comprises two orders, with the order Urostylida exhibiting a polyphyletic distribution across two distinct lineages: (1) *Pseudokeronopsis erythrina* forms a sister clade with nine *Euplotes* species (Euplotida) and *Bakuella subtropica* (61% ML, 0.84 BI); (2) *Anteholosticha monilata* clusters as sister to Sporadotrichida species.

### 3.4 Secondary Structures of nSSUr‐V4 and ITS2

**FIGURE 10:**
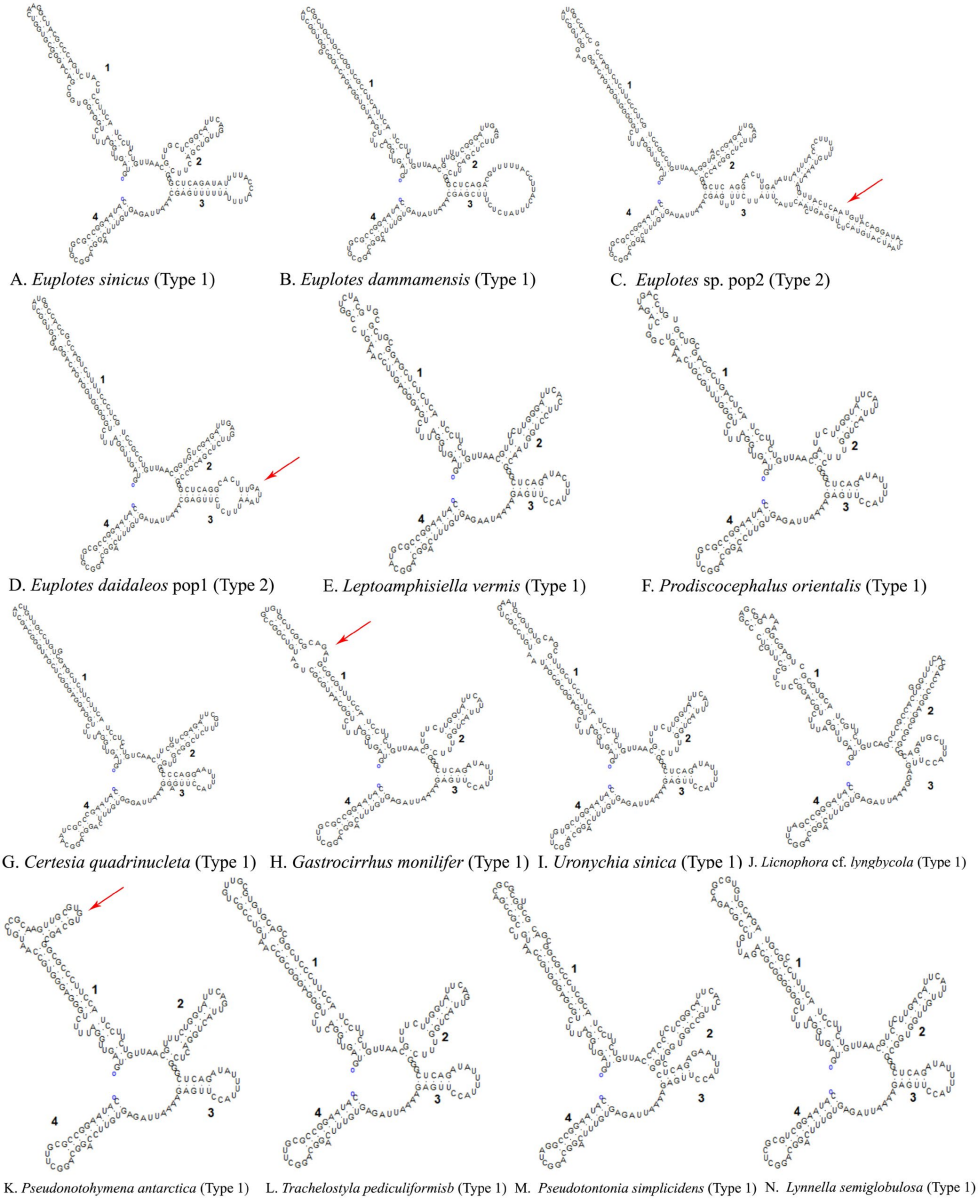
Putative secondary structures of nSSU‐V4 for selected species in this study. The red arrow indicates a significant difference. For remaining species, see Figure S1.

**FIGURE 11:**
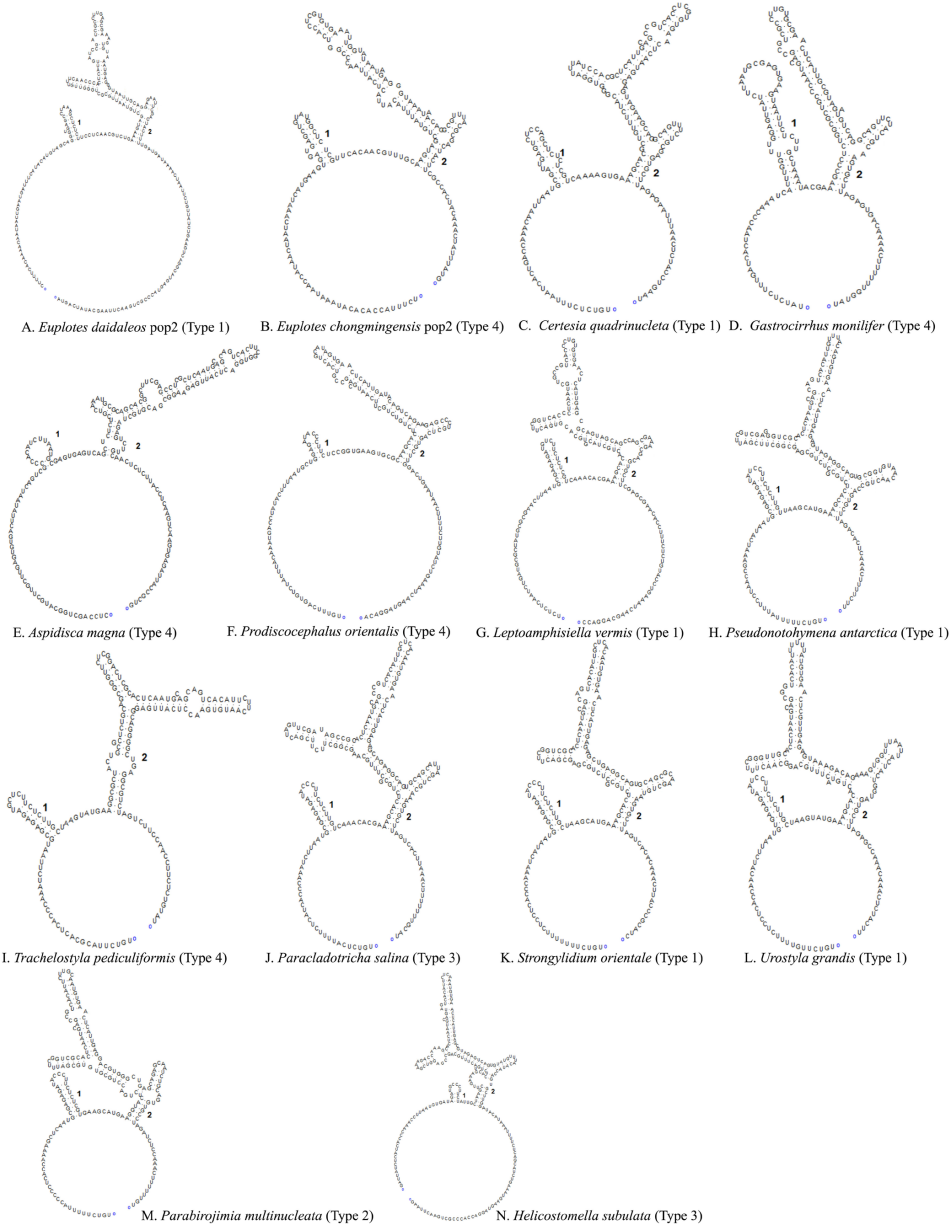
Putative secondary structures of IST2 for selected species in this study. For remaining species, see Figure S2.

**FIGURE 12:**
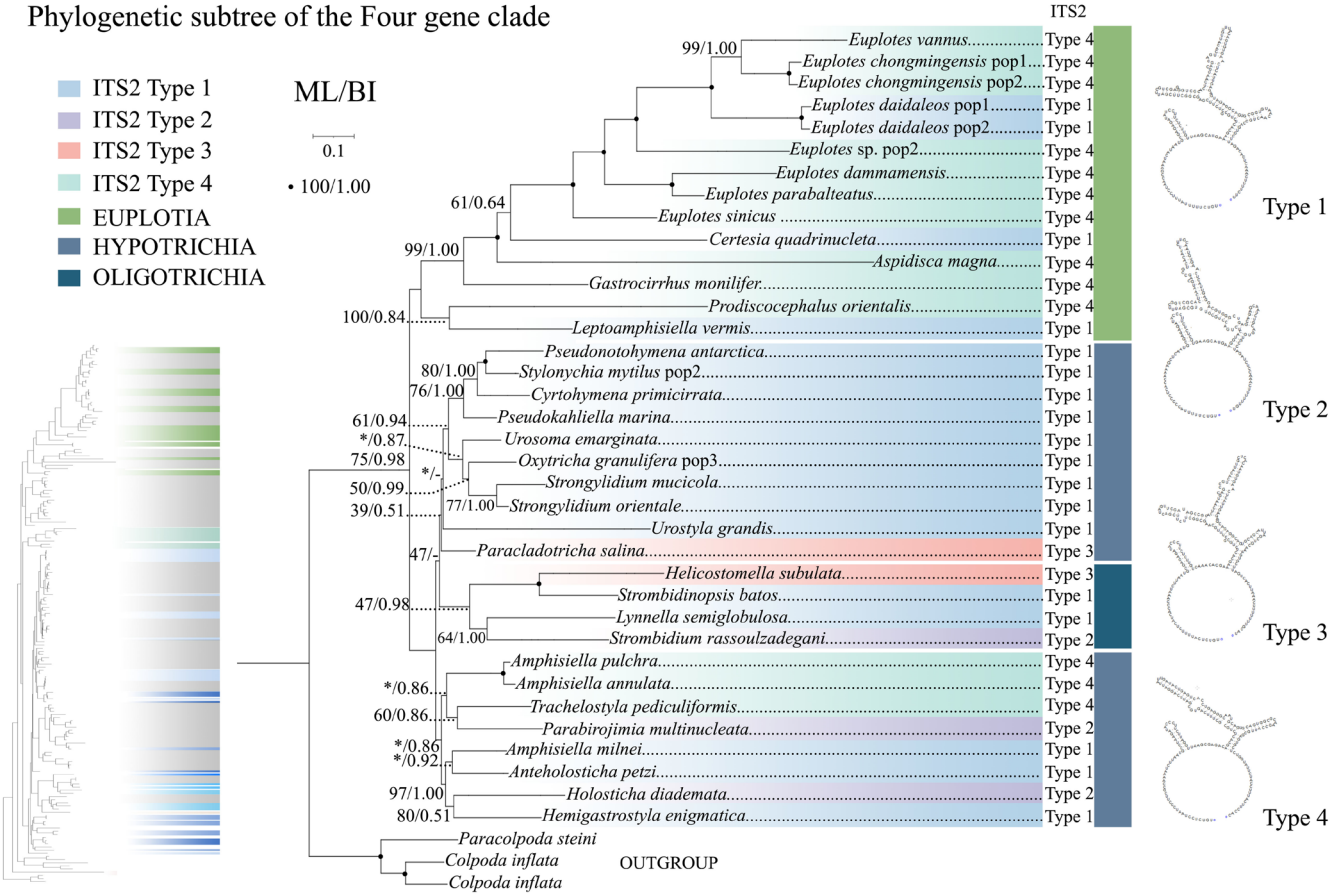
The subtree (right) includes only taxa with predicted ITS2 secondary structures, extracted from the full phylogenetic context (left). Branches are colored by ITS2 type (1–4), with nodal support values given as ML/BI. Species are labeled with their ITS2 type (1–4), corresponding to the structural models depicted on the right.

**FIGURE 13:**
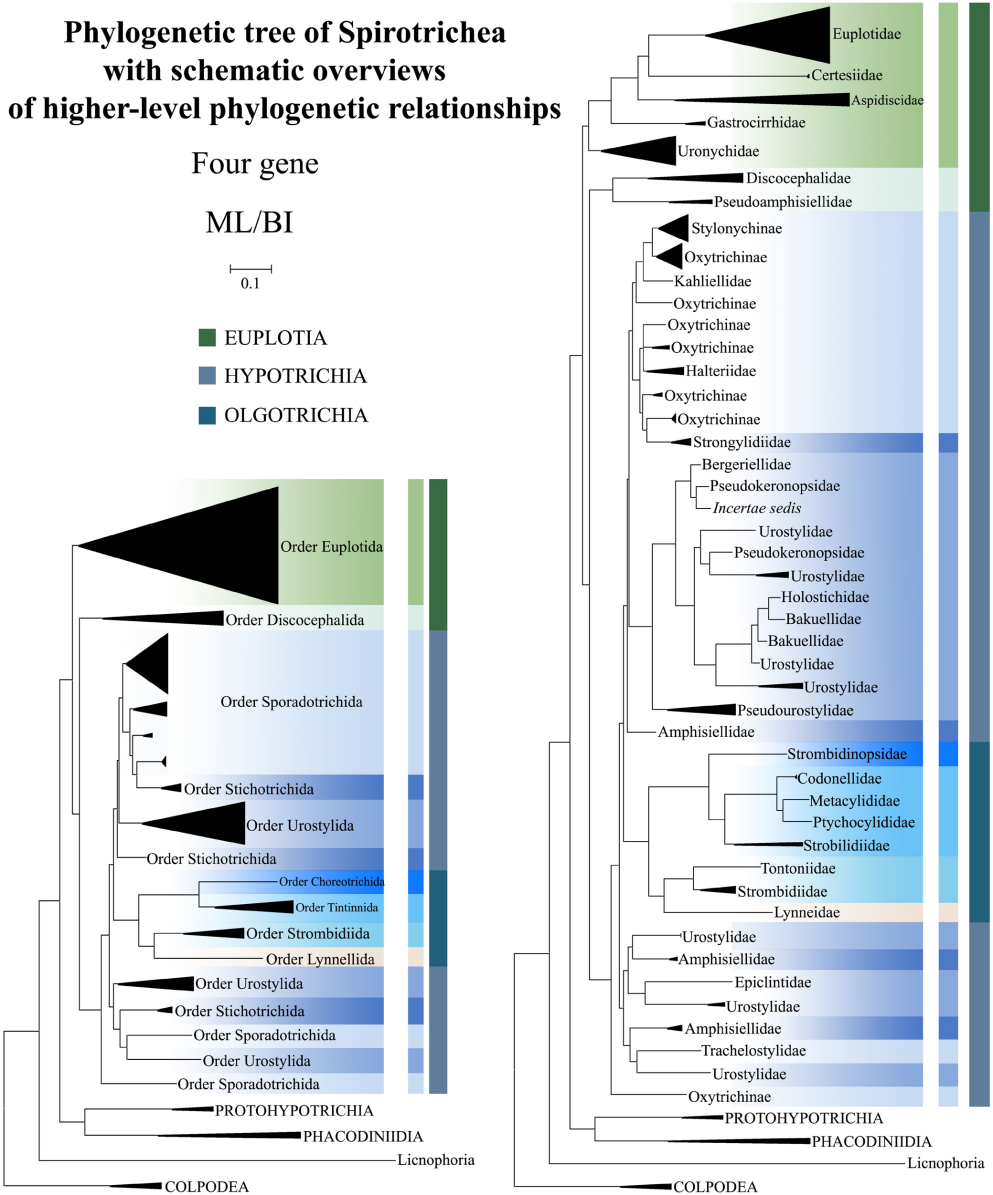
The schematic trees of phylogenetic relationships within the class Spirotrichea (as depicted in Figures 1 and 2) illustrating major taxonomic groups at or above the family level. To clarify the overall phylogenetic structure, schematic trees summarizing relationships at the order level and family level are presented on the left and right, respectively.

Secondary structures of the nSSUr‐V4 and ITS2 regions were predicted and comparatively analyzed across 46 representative species of the class Spirotrichea. Specifically, 46 distinct nSSUr‐V4 secondary structures and 34 ITS2 secondary structures were computationally modeled (Figures 10 and 11, Figures S1 and S2). This taxon sampling comprehensively covers all major subclasses (Euplotia, Hypotrichia, Oligotrichia, Licnophoria) and their constituent orders.

Consensus secondary structure prediction for the nSSUr‐V4 region reveals a conserved architectural pattern across Spirotrichea species, characterized by a large loop separated by four helices. Euplotia exhibits pronounced plasticity in helix 4. Within the subclass Euplotia, two dominant secondary structure patterns emerge: Type 1, which features a large terminal hairpin loop within helix 4; Type 2, which exhibits an extended helical segment in helix 4 with the presence of 1–2 additional helices. The families Certesiidae, Aspidiscidae, Gastrocirrhidae, and Uronychidae uniformly exhibit the Type 1 structure pattern (single large hairpin loop in Helix 4) (Figure 10). In contrast, Euplotidae demonstrates two structure patterns: majority species (*Euplotes* spp.) adopt the Type 2 configuration characterized by a single accessory helix in helix 4. *Euplotes* sp. pop2 represents a distinct variant of Type 2 with two accessory helices (Figure 10). Four species, *E. minuta* , *E*. *parabalteatus*, *E*. *dammamensis*, and *E*. *sinicus*, retain the ancestral Type 1 architecture (Figure 10, Figure S1). Within the order Discocephalida, structural divergence between *Prodiscocephalus orientalis* (Discocephalidae) and *Leptoamphisiella vermisis* (Pseudoamphisiellidae) is predominantly localized to helix 1 of the nSSUr‐V4 region, characterized by: interior loop remodeling and hairpin loop reconfiguration (5′‐CGGUCAGAUGACCUGU‐3′ vs. 5′‐CCGGUCUACGUGC‐3′) (Figure 10). Hypotrichia and Licnophoria display conserved helix 4 configurations, with primary structural variations concentrated in helix 1, characterized by terminal helix extensions incorporating additional helices or hairpin loops. Within the subclass Hypotrichia, *Pseudonotohymena antarctica* exhibits a different structure, characterized by a 14‐nucleotide hairpin loop appended to the terminus of helix 1. Within the subclass Oligotrichia, the nSSU‐V4 secondary structure exhibits high evolutionary conservation, with *Pseudotontonia simplicidens* demonstrating a unique structural deviation: the absence of unpaired spacer nucleotides between Helix 2 and Helix 3, contrasting with the conserved 5′‐GGG‐3′ spacer motif observed in other oligotrich species (Figure 10).

The ITS2 secondary structure reveals a conserved architectural pattern across Spirotrichea species, characterized by a central loop separated by two helices. Helix 1 is shorter than helix 2 (Type 1), which can be split into three or five parts. Compared to the Type 1, other secondary structures show unique structural complexities: contain extra bulge loops in helix 2 (Type 2 and Type 3) or shows a simplified three‐part division of helix 2 (Type 4) (Figure 11). Collectively, the majority of *Euplotes* species predominantly exhibit the Type 4 (Figure 12, Figure S2). However, *Euplotes daidaleos* belong to Type 1 (Figures 11 and 12). Within the order Discocephalida, *Prodiscocephalus orientalis* (Discocephalidae) exclusively adopts the Type 4, while *Leptoamphisiella vermis* (Pseudoamphisiellidae) exhibits the Type 1 (Figures 11 and 12). Within the subclass Hypotrichia, the three orders Sporadotrichida, Stichotrichida, and Urostylida each exhibit distinct structural patterns in their rRNA secondary structures. These order‐level structural manifest as: (1) Sporadotrichida is mainly Type 1, with only *Trachelostyla pediculiformis* showing Type 4; (2) Stichotrichida contains three types of structures: Type 1, Type 3 and Type 4 patterns; (3) Urostylida contains two structural Types 1 and 2 patterns. Most Oligotrichia species have the same pattern Types 1 and 2 structures as those with fewer bulge loops, while *Helicostomalla subulata* has the pattern with more bulge loops (Type 3) (Figure 11, Figure S2).

## 4 Discussion

### 4.1 Re‐Identifying Phylogenetic Relationships Within Class Spirotrichea Through New Sequences

As disclosed in prior studies (REF), the current research has uncovered that individual gene trees display varied and unclear clustering patterns. For example, the SSU‐rRNA gene tree shows that the order Discocephalida is part of the subclass Euplotia (Figure 3), whereas the ITS1‐5.8S‐ITS2 tree exhibits the order Discocephalida within the Hypotrichia (Figures 5 and 6). Moreover, single gene trees might display diverse clustering patterns across different studies, whereas the multiple gene trees exhibit almost identical topologies (Gao et al. 2016; Jiang et al. 2024; Lian et al. 2023; Sheng et al. 2018; Wang et al. 2021; Yi and Song 2011). All these verify the importance of utilizing multi‐gene trees to illuminate relationships within Spirotrichea. In this research, a wider range of species was sampled to refine the topologies of multi‐gene trees.

Here, a total of 94 new sequences covering two spirotrichean subclasses (Euplotia and Hypotrichia) were acquired, and multi‐gene trees including three nuclear genes and one mitochondrial gene are rebuilt. Our phylogenetic reconstructions yield topological configurations congruent with previously published multi‐gene trees. The subclass Oligotrichia is nested within Hypotrichia, thereby rendering Hypotrichia non‐monophyletic (Fernandes and Schrago 2019; Jiang et al. 2024; Yi and Song 2011). Three Hypotrichia lineages are evolutionarily distant from the majority of Hypotrichia species, and Oligotrichia is located between them. Notably, this phylogenetic incongruence is not a recent methodological artifact. As documented by Yi and Song (2011), the subclass Hypotrichia was resolved as non‐monophyletic, fragmented by the interposition of Oligotrichia into group 1 (the majority of hypotrich species) and group 2 (*Pseudokeronopsis* + *Holosticha*). Following expanded taxon sampling, the genus *Pseudokeronopsis* is now resolved as a member of the family Pseudourostylidae, clustering with core Hypotrichia lineages (Gao et al. 2016). Additionally, novel lineages have been incorporated into Group 2, including eight genera (Jiang et al. 2024 and this study). Within the three orders Sporadotrichida, Stichotrichida, and Urostylida, the clustering patterns of 13 newly sequenced species further confirm the paraphyly of orders Sporadotrichida, Stichotrichida, and Urostylida (Gao et al. 2016; Jung et al. 2019; Zhang et al. 2022). For *Euplotes* species, the polyphyletic tree (Figure 1) maintained its five‐clade topology after the addition of five newly sequenced species (Lian et al. 2023). Notably, the clustering of *Strombidium* cf. *sulcatum* with the Euplotida in our multi‐gene tree is considered anomalous and likely an analytical artifact. This is because the available data for this species are limited to a single, relatively short mtSSU rRNA gene sequence. This combination of highly limited data availability for the species and the fast evolutionary rate of the mtSSU rRNA gene likely resulted in a Long‐Branch Attraction (LBA) artifact (Quince et al. 2017; Wang et al. 2017). Therefore, the position of *Strombidium* cf. *sulcatum* should be interpreted with caution and is not considered robust evidence for a phylogenetic relationship. Consequently, our discussion of the core relationships within the clade remains unaffected.

### 4.2 Reconstructing Euplotida Evolution: Morpho‐Molecular Synapomorphies Resolve Deep Phylogenetic Conflicts

The subclass Euplotia currently comprises two orders and seven families, with the order Euplotida subdivided into five constituent families (Euplotidae, Aspidiscidae, Certesiidae, Gastrocirrhidae, and Uronychidae). The family Uronychidae resolves into two clades: *Diophrys* + *Pseudodiophrys* and *Uronychia* + *Apodiophrys* + *Paradiophrys*. Within the order Euplotida, the families Euplotidae and Certesiidae are sister groups. The family Uronychidae was resolved as sister to all other families in the order (Figures 1, 3, 5, and 7). This phylogenetic inference is robustly corroborated by morphological, habitat profile, and rRNA secondary structure patterns across lineages. Morphologically, the genus *Diophrys* retains two distinct undulating membranes (Kwon and Shin 2006). In contrast, Uronychia exhibits a different ciliary pattern, losing the endoral membrane while retaining a single paroral membrane. Notably, the evolutionary loss of the endoral membrane extends beyond Uronychia, characterizing four additional families (Euplotidae, Aspidiscidae, Certesiidae, and Gastrocirrhidae) (Shen et al. 2009; Lian et al. 2023). Concomitantly, the family Uronychidae retains distinct left marginal cirri, a plesiomorphic trait absent in Euplotidae and Aspidiscidae (Lynn 2008). We endorse the hypothesis advanced by Lian et al. (2023) that progressive evolutionary degeneration of left marginal cirri occurred across descendant lineages. Habitat delineates clear eco‐evolutionary boundaries: Uronychidae, Certesiidae, and Gastrocirrhidae exclusively inhabit marine environments, and Euplotidae and Aspidiscidae occupy both marine and freshwater biotopes.

Regarding the secondary structure of sequences, the nSSU‐V4 secondary structures of Aspidiscidae, Certesiidae, Gastrocirrhidae, and Uronychidae all exhibit the relatively ancestral Type 1 configuration (Lee and Gutell 2012). In contrast, a novel nSSU‐V4 structural pattern (Type 2) emerges within the Euplotidae family (Figure 10, Figure S1). Based on rRNA secondary structure patterns, we delineate Euplotes into five phylogenetically coherent clades (Clades 1–5), contrasting with the six‐clade topology proposed by Wardani et al. (2025). We consolidated Clades 5 and 6 proposed by Wardani et al. (2025) into a unified lineage, based on structural profiling revealing that *Euplotes vannus* and *E. crassus* exhibit Type 2 secondary structures in their nSSU‐V4 regions (Figure S2). ITS2 secondary structures exhibit evolutionarily conserved patterns, with basally diverging lineages, including *Gastrocirrhus monilifer* (Gastrocirrhidae), *Euplotes sinicus* (Clade 5), and *E*. *dammamensis* (Clade 4), convergently exhibiting Type 4 structural configurations (Figures 1 and 11, Figure S2). A pattern of structural variation in *Euplotes* ITS2, which parallels the variation reported for nSSU‐V4 (Wardani et al. 2025), is evident. This is exemplified by the presence of the complex, multi‐helix structure (Type 1) in the clade containing *E. daidaleos*, while other clades possess the simpler tripartite Type 4 (Figure 12). Nevertheless, the evolutionary direction of this change remains unclear without a firmly established ancestral state. Our phylogenomic analyses resolve Certesiidae as the sister family to Euplotidae, contrasting with the Aspidiscidae–Euplotidae sister relationship proposed by Lian et al. (2023). However, morphological analyses reveal a striking similarity between Aspidiscidae and Euplotidae, specifically in the extreme reduction of the paroral membrane, a condition that contrasts with the oral ciliature retained by Certesiidae (Lynn 2008). Concurrently, these families exhibit niche conservatism, with Aspidiscidae and Euplotidae occupying both marine and freshwater biotopes. Notably, the pattern of ITS2 structural variation across the order Euplotida bears a resemblance to the variation pattern of nSSU‐V4 observed by Wardani et al. (2025) in the family Euplotidae. Basal branches of Aspidiscidae and Euplotidae display structurally simpler Type 4 patterns, but Certesiidae retains a more complex Type 1 architecture (Figures 11 and 12, Figure S2). Aspidiscidae is stably resolved as sister to a clade containing Euplotidae and Certesiidae, receiving maximum support (100% ML, 1.00 BI). Based on the phylogenetic evidence presented above, we propose that Certesiidae and Euplotidae are sister clades.

### 4.3 Resolving Hypotrichia‐Oligotrichia Phylogeny: Ecological Drivers and Structural Synapomorphies

The subclass Hypotrichia represents one of the most morphologically diverse lineages within ciliates, yet its phylogeny remains contentiously unresolved despite extensive study (Berger 2007; Berger 2011; Luo et al. 2021). Over a dozen proposed phylogenetic frameworks, including those of Corliss (1979), Lynn (2008), Sheng et al. (2018) and Song et al. (2023), exhibit persistent topological incongruence. This taxonomic impasse stems from a persistent incongruence between morphological and molecular phylogenetic frameworks (Gentekaki et al. 2014; Huang et al. 2012; Lynn 2008). Based on ventral ciliary patterns and partial morphogenetic characteristics, Lynn (2008) recognized three orders: Stichotrichida, Urostylida, and Sporadotrichida. However, in numerous studies, each order has been found to be non‐monophyletic. The three orders are nested within each other (Gao et al. 2016; Huang et al. 2016; Zhang et al. 2022). Nevertheless, this ambiguous phylogenetic structure is being progressively resolved through extended taxonomic sampling. The greatest topological resolution was achieved within the order Sporadotrichida. In Gao et al. (2016), Sporadotrichida was divided into eight clades, whereas the present study has reduced this number to four. The current topology consists of a clade represented by Stylonychinae + Oxytrichinae and three separate lineages represented by *Pseudocyrtohymena koreana*, *Trachelostyla pediculiformis*, and *Hemigastrostyla enigmatica*, respectively. Phylogenetic analyses resolve Urostylida into a core clade and several peripheral lineages, corroborating prior taxonomic frameworks. The non‐core taxa consistently branch outside the core group (Li et al. 2009; Lyu et al. 2018; Zhao et al. 2015). Meanwhile, consistent with Li et al. (2009), Stichotrichida consistently branches as sister to Urostylida, despite their considerable morphological divergence. The ventral cirri in Stichotrichida are arranged as one or more longitudinal files of varied lengths with linear organization, whereas in Urostylida they exhibit a distinctly serrated (zigzag) pattern (Lynn 2008). Additionally, the family Strongylidiidae (Stichotrichida) occupies a peripheral position within Sporadotrichida, further blurring the taxonomic demarcation between these orders (Figure 2). Despite the application of secondary structure analyses, the three orders remain phylogenetically inseparable. We posit that the morphological criteria currently used to delineate these orders may not hold up under molecular scrutiny (Berger 2007; Gao et al. 2016; Yi and Song 2011). Despite persistent non‐monophyly of the three hypotrich orders (Stichotrichida, Urostylida, Sporadotrichida), we uncover a robust correlation between habitat transitions and phylogenetic divergence. Sporadotrichida inhabit marine, freshwater, and terrestrial environments. Within Urostylida, the core group includes freshwater and terrestrial species (e.g., Pseudourostylidae), while other taxa such as *Epiclintes* (marine) and *Holosticha* (freshwater to brackish) show distinct habitats (Jung et al. 2019; Lynn 2008). This ecological distribution aligns with their positions in multi‐gene phylogenetic trees, suggesting a terrestrial invasion process from marine environments among non‐core Urostylida lineages. This finding indicates that terrestrial invasion served as a key speciation driver, in which ecological adaptation triggered cladogenesis.

Our findings robustly corroborate prior phylogenetic reconstructions, demonstrating that the subclass Oligotrichia is consistently nested as a basal subclade within Hypotrichia across all gene trees analyzed (Gao et al. 2016; Jiang et al. 2024; Yi and Song 2011). The persistent systematic controversy likely stems from both shared genomic conservation between Oligotrichia (e.g., *Strombidium sulcatum* ) and Hypotrichia (e.g., *Uroleptopsis citrinus*) and concerted evolutionary trajectories in their morphological evolution (Sheng et al. 2018; Song et al. 2023). A notable example of this is the ventral girdle kinety in Oligotrichia, which traces back to the complex ventral ciliature of Hypotrichia (Song et al. 2023). The evolutionary divergence of Oligotrichia and Hypotrichia is hypothesized to stem from a common multikinetid‐equipped ciliate ancestor, which subsequently radiated via distinct adaptive pathways: structural simplification of ciliary patterns (Oligotrichia) and functional diversification of cirral systems (Hypotrichia) (Sheng et al. 2018; Song et al. 2023). Within the subclass Oligotrichia, the phylogenetic placement of the order Lynnellida exhibits persistent incongruence across gene trees: (1) sister to Tintinnida in multi‐gene and LSU‐rRNA gene trees and sister to Strombidiida in SSU‐rRNA gene tree (Gao et al. 2016); (2) sister to Tintinnida in multi‐gene and SSU‐rRNA gene trees and sister to Strombidiida in ITS1‐5.8 s‐ITS2, LSU‐rRNA gene trees (Song et al. 2023); (3) sister to Strombidiida in SSU‐rRNA gene tree (Liu et al. 2015); (4) sister to Tintinnida in SSU‐rRNA gene tree (Liu et al. 2011). Contrastingly, our study resolves Lynnellida as a consistent sister clade to Strombidiida across: multi‐gene, SSU‐rRNA gene, and LSU‐rRNA gene trees. Morphologically, Lynnellida and Strombidiida share similarities in their AZM and somatic ciliary patterns, yet Lynnellida resembles Tintinnida in the absence of differentiation between anterior and ventral membranelles (AM/VM) (Liu et al. 2011, 2015). Furthermore, comparative analysis of nSSU‐V4 secondary structures reveals that *Lynnella semiglobulosa* is similar to Strombidiida species; this pattern holds true for both order Strombidiida (e.g., *Pseudotontonia simplicidens*) and order Tintinnida (e.g., *Strombidium rassoulzadegani*) (Figure 10, Figure S1). Based on this structural synapomorphy, we provisionally assign *L*. *semiglobulosa* to a basal position within Strombidiida, hypothesizing its status as a transitional taxon bridging morphological and molecular data between the orders Tintinnida and Strombidiida.

### 4.4 Resolving Long‐Standing Controversies: Licnophoria as Basal Lineage and Discocephalida as Key Evolutionary Transition

The phylogenetic position of Licnophora has long been contentious. While some studies placed it outside the Spirotrichea (Chen et al. 2015; Gao and Katz 2014), our integrated analysis provides clarity through two key advances. SSU rRNA gene sequences and nuclear replication band characteristics consistently place Licnophora within the clade Spirotrichea (Lynn and Strüder‐Kypke 2002; Lian et al. 2023; this study). Discovery of diagnostic structural markers: We identified conserved nSSU‐V4 secondary structures (e.g., identical Helix 2–3 spacer motifs) shared exclusively between Licnophoria and Euplotia (Figure 10, Figure S1), providing unambiguous evidence for their common ancestry within Spirotrichea. Multi‐gene phylogenies consistently position Licnophoria at the base of Spirotrichea (Gao et al. 2016; this study). However, some phylogenetic reconstructions reject the placement of Licnophora within Spirotrichea (Chen et al. 2015; Gao and Katz 2014; Miao et al. 2009). Based on novel nSSU rRNA V4 structural evidence, we support the proposal that Licnophoria represents an early‐diverging, unique branch of Spirotrichea (Gao et al. 2016). The basal status of Licnophoria redefines the root of Spirotrichea evolution and establishes nSSU‐V4 structures as critical tools for identifying early‐branching taxa. Due to limited taxon sampling and inconsistent nodal support, Licnophoria is provisionally designated as *incertae sedis*.

The position of Discocephalida appears unstable, with several proposed locations: (1) between Euplotida and Hypotrichia, forming a deep‐branching clade (Miao et al. 2011); (2) at the base of Euplotida (Gao et al. 2016); (3) at the base of Hypotrichia (Lian et al. 2023; this study). Despite morphological links to Euplotida (e.g., discoid head), discocephalids exhibit conflicting molecular placements. We reconcile this paradox through a novel evolutionary model based on three lines of evidence: (1) Transitional morphology: The discoid head of Discocephalida links it to Euplotida, while the number of fronto‐ventral‐transverse cirral anlagen distinguishes them (Lian et al. 2023; Shao et al. 2008). Furthermore, they share similar ciliary patterns. For instance, the number of FVT‐circular primordia (Miao et al. 2011) provides evidence for mosaic evolution. (2) Molecular signature: The ITS2 secondary structure pattern of Discocephalida (*Prodiscocephalus orientalis*) also exhibits similarities with basal groups of Euplotida and *Trachelostyla pediculiformis* (a member of Hypotrichia), classified as Type 4 (Figure 11), confirming transitional status. (3) Phylogenetic placement: Multi‐gene trees robustly position Discocephalida at the Hypotrichia base (Lian et al. 2023; this study), acting as a bridge between subclasses. Collectively, these findings position Discocephalida as a pivotal evolutionary transitional taxon, whose morphological mosaicism offers a paradigm for studying trait diversification in ciliate evolution, and which likely represents a transitional lineage branching between Euplotida and Hypotrichia.

## Author Contributions

**Bailin Li:** formal analysis (lead), software (lead), writing – original draft (lead), writing – review and editing (equal). **Chunyu Lian:** formal analysis (equal), investigation (equal), software (equal). **Yongqiang Liu:** formal analysis (equal), software (equal). **Zhongming Wang:** formal analysis (supporting), software (equal). **Xuming Pan:** conceptualization (equal), funding acquisition (lead), methodology (lead), supervision (lead), writing – review and editing (equal). **Li Wang:** conceptualization (equal), methodology (equal), supervision (equal).

## Funding

This work was supported by the National Natural Science Foundation of China (project numbers: 32270544, 32570595, 32500386), Outstanding Youth Program of Natural Science Foundation of Heilongjiang Province (Grant No. YQ2023C033), and Doctoral Innovation Fund of Harbin Normal University (HSDBSCX2025‐12).

## Disclosure

Study Registration: This study was pre‐registered on the Open Science Framework. The pre‐registration can be accessed via the following URL: https://osf.io/zumyr.

## Conflicts of Interest

The authors declare no conflicts of interest.

## Supporting information


**Figure S1:** The putative secondary structures of nSSU‐V4 in the present study.

**Figure S2:** The putative secondary structures of ITS2 in the present study.

## Data Availability

The data presented in the study is deposited in the NCBI database (https://www.ncbi.nlm.nih.gov/) repository; accession numbers, lengths, and G&C contents are shown in Table 4.
