# Supplementary figures and images for: A Multigene Perspective on the Phylogeny and Evolutionary Relationships of Class Spirotrichea (Ciliophora)
Source: Ecol Evol. 2025 Dec 16;15(12):e72668. doi: 10.1002/ece3.72668 (PMC12706529; doi:10.1002/ece3.72668)

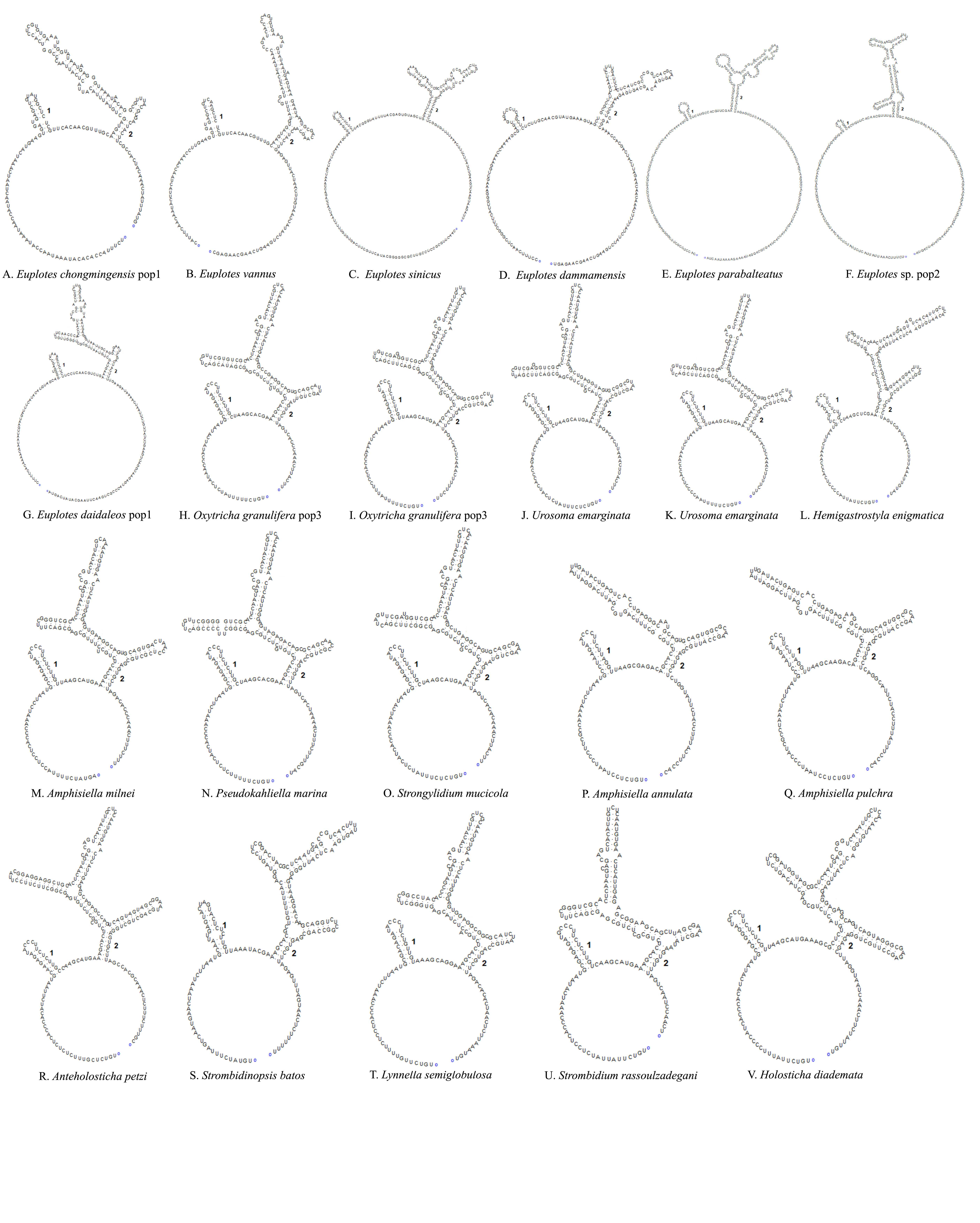

Supplement: Supplementary file 1 — Figure S1: The putative secondary structures of nSSU‐V4 in the present study. [file ECE3-15-e72668-s002.jpg]

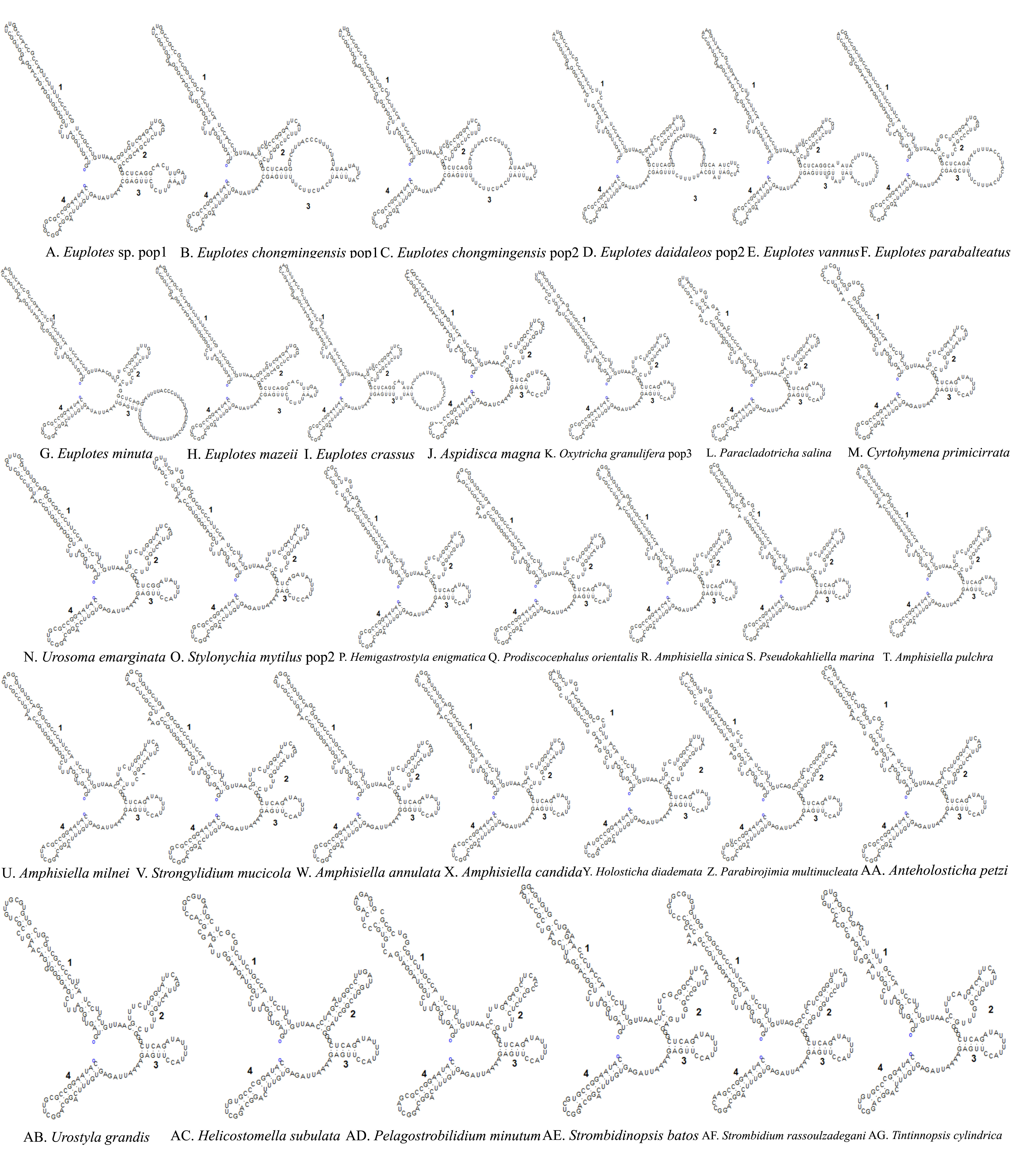

Supplement: Supplementary file 2 — Figure S2: The putative secondary structures of ITS2 in the present study. [file ECE3-15-e72668-s001.jpg]
